# Comparative cardiovascular risk of sulfonylureas with low‐ and high‐affinities for cardiac mitochondrial adenosine triphosphate‐sensitive potassium channels versus dipeptidyl peptidase‐4 inhibitors in patients with type 2 diabetes: A cohort study

**DOI:** 10.1111/dom.70157

**Published:** 2025-09-29

**Authors:** Mei‐Hsiu Chen, Liang‐Yu Lin, Tung‐Ying Hung, Tzu‐Chieh Lin, Chia‐Yu Li, Tzu‐Han Lin, Yi‐Hsuan Chen, Jun‐Ting Liou, Meng‐Ting Wang

**Affiliations:** ^1^ Division of Endocrinology, Department of Internal Medicine Far Eastern Memorial Hospital New Taipei City Taiwan; ^2^ Department of Biomedical Engineering Ming Chuang University Taoyuan City Taiwan; ^3^ Faculty of Medicine National Yang Ming Chiao Tung University Taipei Taiwan; ^4^ Division of Endocrinology and Metabolism, Department of Medicine Taipei Veterans General Hospital Taipei Taiwan; ^5^ Department of Pharmacy National Yang Ming Chiao Tung University Taipei Taiwan; ^6^ Division of Cardiology China Medical University Hsinchu Hospital Zhubei City Taiwan

**Keywords:** cardiac mitochondrial adenosine triphosphate‐sensitive potassium channels, cohort study, dipeptidyl peptidase‐4 inhibitors, major adverse cardiovascular events, sulfonylureas, type 2 diabetes mellitus

## Abstract

**Aims:**

To individually evaluate the cardiovascular safety of low‐ and high‐affinity cardiac mitochondrial ATP‐sensitive potassium (mitoK_ATP_) channel sulfonylureas by comparing each to dipeptidyl peptidase‐4 inhibitors (DPP‐4i), a generally cardiovascular‐neutral comparator, given prior evidence suggesting greater cardiovascular risk with high‐affinity agents and the absence of a neutral active comparator.

**Methods:**

A new user, active comparator cohort study using propensity score‐based inverse probability of treatment weighting was conducted with Taiwan's nationwide claims database (2012–2022). Patients with recent pre‐entry cardiovascular events were excluded. The primary outcome was 3‐point major adverse cardiovascular events (MACE: ischaemic stroke, myocardial infarction, cardiovascular death); secondary outcomes included each MACE component and all‐cause mortality.

**Results:**

The study cohort included 466 158, 83 031, and 473 539 new users of low‐affinity mitoK_ATP_‐channel sulfonylureas (gliclazide, glimepiride), high‐affinity sulfonylureas (glyburide, glipizide), and DPP‐4i, respectively (mean age, 60.2 years; 55.6% male). Compared with DPP‐4i, low‐affinity sulfonylureas were not associated with increased risks of 3‐point MACE (Hazard ratio [HR], 1.01; 95% confidence interval [CI], 0.96–1.06), cardiovascular death (HR, 1.01; 95% CI, 0.93–1.08), or all‐cause mortality (HR, 0.97; 95% CI, 0.93–1.01). Conversely, high‐affinity sulfonylureas were associated with 1.17–1.31‐fold increased risks of 3‐point MACE, cardiovascular death, and all‐cause mortality.

**Conclusions:**

Initiating low‐affinity mitoK_ATP_‐channel sulfonylureas demonstrated cardiovascular safety comparable to DPP‐4i, whereas high‐affinity sulfonylureas were linked to increased cardiovascular risk and all‐cause mortality. These findings suggest low‐affinity sulfonylureas are a safer alternative to high‐affinity agents and as safe as DPP‐4i. They may be appropriate for individuals with diabetes when newer therapies are inaccessible or unnecessary.

## 1 INTRODUCTION

Diabetes mellitus poses a major global health burden,^1^ with significant regional disparities in type 2 diabetes management, particularly in the affordability and accessibility of antidiabetic medications.^2^ Sulfonylureas, in use for more than five decades, remain widely prescribed due to their glucose‐lowering efficacy, affordability, and broad availability, making them especially common in developing or low‐income countries.^2^, ^3^ Despite the introduction of newer antidiabetic agents,^4^ sulfonylureas continue to be extensively used worldwide,^5^, ^6^ particularly in diabetic patients without cardiovascular or renal diseases.

Over the past five decades, the cardiovascular safety of sulfonylureas has been debated, beginning with increased cardiovascular mortality observed with tolbutamide use in the University Group Diabetes Program (UGDP).^5^ Prior research yielded inconsistent results, primarily due to the criticisms regarding comparator selection and the absence of rigorous study designs.^6^ Multiple observational studies^7^, ^8^, ^9^ compared sulfonylureas with metformin, an agent with debated cardiovascular benefits.^10^, ^11^ Most previous studies exhibited significant design‐related biases, including time‐lag bias^12^, ^13^, ^14^ and selection bias,^15^, ^16^ potentially leading to spurious findings. While a few pivotal randomised controlled trials (RCTs) have assessed sulfonylureas against comparators other than metformin,^17^, ^18^, ^19^, ^20^ their generalizability to real‐world settings may be constrained. This limitation may stem from cardiovascular events being analysed in underpowered subgroup analyses.^17^ Additionally, half of comparator groups' patients received other sulfonylureas,^18^ and participants had high cardiovascular risk.^19^ These factors could substantially limit the applicability of the findings.

The high affinity of certain sulfonylureas for cardiac mitochondrial adenosine triphosphate‐sensitive potassium (mitoK_ATP_) channels has been identified as a primary contributor to sulfonylurea‐associated adverse cardiovascular outcomes.^21^ Previous studies indicated that high‐affinity sulfonylureas are associated with an increased risk of major adverse cardiovascular events (MACE) compared to low‐affinity sulfonylureas.^22^, ^23^ However, in the absence of an active comparator generally regarded as cardiovascularly neutral, such as DPP‐4 inhibitors, it remains unclear whether low‐affinity sulfonylureas exert neutral effects and whether high‐affinity sulfonylureas increase cardiovascular risk in patients with diabetes.

This study aimed to provide real‐world evidence on the comparative cardiovascular safety of low‐affinity cardiac mitoK_ATP_ channel sulfonylureas and DPP‐4 inhibitors, as well as high‐affinity sulfonylureas versus DPP‐4 inhibitors, in a nationwide population of patients with type 2 diabetes.

## 2 METHODS

### 2.1 Study design and data sources

We conducted a cohort study with a new‐user design, active comparator, and inverse probability of treatment weighting (IPTW) based on propensity scores,^24^, ^25^, ^26^ using data from Taiwan's National Health Insurance Research Database (NHIRD) from 1 January 2012 to 31 December 2022. The NHIRD provides comprehensive data on medical diagnoses, procedures, and prescription refill records of outpatient, emergency, and inpatient visits, covering over 99% of 23 million residents in Taiwan.^27^ The NHIRD was linked with the National Death Registry records to obtain the death records. Multiple disease codes, including those for cardiovascular diseases, have been validated with high accuracy in the NHIRD,^27^ which has also been used to evaluate the effectiveness and safety of antidiabetic medications.^28^, ^29^

### 2.2 Study population

The study cohort comprised type 2 diabetic patients initiating a sulfonylurea agent or a DPP‐4 inhibitor from 1 January 2013 to 31 December 2021. Specifically, the eligible study cohort included diabetic patients, defined as ≥2 outpatient visits or one inpatient visit for diabetes in a given year.^30^, ^31^ Among these patients, we identified new users of cardiac mitoK_ATP_ channels low‐, high‐affinity sulfonylureas, or DPP‐4 inhibitors. New users were defined as having no prescription refill records of both sulfonylureas and DPP‐4 inhibitors in the prior year. Cohort entry was defined as the date of the first filled prescription of either of the two study drugs. Eligible patients were aged ≥18 years at cohort entry. We excluded patients receiving two types of sulfonylureas or DPP‐4 inhibitors in combination with sulfonylureas at cohort entry. Patients with any diagnoses of type 1 diabetes mellitus, less than 1‐year continuous NHI enrolment, or pregnancy in the prior year before cohort entry were excluded. Additionally, we excluded patients hospitalised for acute myocardial infarction (MI), coronary revascularization, unstable angina, heart failure, transient ischemic attack, ischemic or haemorrhagic stroke within 90 days before the cohort entry date. Further definitions of inclusion and exclusion criteria are detailed in Table S1.

The study cohort was followed from the cohort entry date until the earliest occurrence of a cardiovascular endpoint (defined below), treatment discontinuation (defined as a >60‐day prescription refill gap), switch to or add‐on to the comparator drug, pregnancy, NHI disenrollment, death, or the end of the study period (31 December 2022).

### 2.3 Outcome definitions

The primary outcome was the 3‐point MACE, including cardiovascular mortality, hospitalisation with a primary diagnosis for MI, and ischaemic stroke. MI and ischaemic stroke outcomes were identified in inpatient settings using validated algorithms with positive predictive values of at least 88%.^32^, ^33^ Cardiovascular death events were ascertained from the National Death Registry records. Secondary outcomes included each MACE component, hospitalisations for heart failure, arrhythmia, or hypoglycaemia, and all‐cause mortality. Further outcome definitions were detailed in Table S1.

### 2.4 Potential confounders and baseline subgroups

According to a thorough literature review and clinical expertise, we considered the following potential confounders: (1) demographics, including age, sex, and calendar year of cohort entry; (2) socioeconomic status based on monthly income‐based insurance premium; (3) proxies of diabetes severity, such as adapted diabetes complication severity index (aDCSI),^20^, ^34^ hypoglycaemia, numbers of antidiabetic medications at cohort entry, types of antidiabetic drugs on cohort entry, and use of antidiabetic medications at baseline; (4) measures of health care utilisation as an indicator for health care intensity; (5) comorbidities (e.g., cardiovascular disease, chronic kidney disease, and chronic obstructive pulmonary disease); and (6) prescription drug usage, such as cardiovascular medications, anti‐inflammatory agents, and anticonvulsants. All confounders were measured in the 12 months before cohort entry, except comedications, which were assessed in the prior 6 months; definitions are provided in Table S1.

We conducted stratified analyses by the following subgroups: (1) presence versus absence of any prior cardiovascular disease, and (2) presence versus absence of prior insulin use.

### 2.5 Sensitivity analyses

We performed several pre‐specified sensitivity analyses to assess the robustness of our main findings (Table S2). We redefined 3P‐MACE to include primary diagnosis or any of the secondary diagnoses of MI or ischemic stroke, excluded patients with prior MI or ischemic stroke hospitalisations within 1 year before cohort entry, and conducted 1‐ and 2‐year intention‐to‐treat analyses, ignoring treatment discontinuation or changes. Continuous treatment was alternatively defined using 30‐day and 90‐day grace periods and repeated analyses excluding stabilised weights exceeding 10.^35^ We further restricted patients with a medication possession ratio ≥0.8^36^ and treated non‐cardiovascular mortality as a competing risk in the 3P‐MACE assessment. To address potential time‐lag bias, we restricted the analysis to patients initiating metformin and subsequently added a sulfonylurea or DPP‐4 inhibitor, controlling for the duration of prior metformin therapy (see Appendix S1). Because hypoglycaemia is linked to cardiovascular risk and may also reflect frailty, we additionally adjusted for severe hypoglycaemia during follow‐up. Hospitalisation for GERD and hypoglycaemia served as negative and positive control outcomes, respectively. E‐values were estimated to quantify the strength of an unmeasured confounder that would need to be associated with both the exposure and the outcomes of interest to fully explain the observed primary association.

### 2.6 Statistical analyses

IPTW using propensity scores was used to adjust for confounding and enable unbiased comparisons across the groups. We estimated the propensity score as the probabilities of receiving low‐affinity, high‐affinity mitoK_ATP_ channel sulfonylureas, and DPP‐4 inhibitors, respectively, using a multinomial logistic regression including all factors listed in Table 1. The estimated weights were stabilised by multiplying the initial weights with the marginal probability of receiving each treatment. We assessed covariate balance among the groups using standardised mean difference before and after weighting, with a magnitude of >0.1 indicating a meaningful difference.^37^

**TABLE 1:** Selected demographic and clinical characteristics of users of MitoK_ATP_ channel‐low affinity sulfonylureas, MitoK_ATP_ channel‐high affinity sulfonylureas, and DPP‐4 inhibitors after propensity score‐based IPTW.

Characteristics^a^	MitoK_ATP_ channel‐low affinity sulfonylureas (*n* = 484 579)	MitoK_ATP_ channel‐high affinity sulfonylureas (*n* = 85 730)	DPP‐4 inhibitors (*n* = 465 396)	MAX aSMD^b^
Age, mean (SD)	60.8 (14.5)	60.7 (14.3)	60.3 (13.8)	0.071
Sex, male No. (%)	268 689 (55.5)	47 451.3 (55.4)	259 771 (55.8)	0.033
Monthly income‐based insurance premium (NTD), No. (%)				
First tertile	200 247.0 (41.3)	35 239.7 (41.1)	189 092.0 (40.6)	0.027
Second tertile	108 785.0 (22.5)	19 312.8 (22.5)	105 757.0 (22.7)	0.027
Third tertile	175 547.0 (36.2)	31 177.1 (36.4)	170 547.0 (36.7)	0.027
Diabetes severity indicators				
aDCSI, mean (SD)	1.0 (1.6)	1.0 (1.5)	0.9 (1.4)	0.050
aDCSI score, No (%)				
0	283 135.0 (58.4)	49 827.4 (58.1)	278 313 (59.8)	0.019
1	79 471.5 (16.4)	14 303.9 (16.7)	77 219.9 (16.6)	0.026
2	53 865.7 (11.1)	9731.9 (11.4)	51 500.6 (11.1)	0.028
3+	68 106.1 (14.1)	11 866.5 (13.8)	58 361.7 (12.5)	0.018
Hypoglycaemia, No. (%)	3931.0 (0.8)	628.8 (0.7)	3273.5 (0.7)	0.025
Diabetes treatment, No. (%)				
Antidiabetics drugs at cohort entry date				
1	117 102.0 (24.2)	21 554.3 (25.1)	113 382.0 (24.4)	0.025
2	283 446.0 (58.5)	50 343.6 (58.7)	279 615.0 (60.1)	0.025
3+	84 030.6 (17.3)	13 831.7 (16.1)	72 398.4 (15.6)	0.024
Diabetes drug on cohort entry date				
Biguanides	327 570.0 (67.6)	57 572.0 (67.2)	319 375.0 (68.6)	0.032
Thiazolidinediones	15 025.4 (3.1)	2699.4 (3.2)	15 262.2 (3.3)	0.048
Alpha‐glucosidase inhibitors	20 989.3 (4.3)	3496.5 (4.1)	19 190.1 (4.1)	0.022
Meglitinides	27 885.5 (5.8)	3586.4 (4.2)	16 498.1 (3.5)	0.022
SGLT2i	7274.1 (1.5)	1345.9 (1.6)	8593.5 (1.9)	0.021
Insulin	70 333.4 (14.5)	11 406.5 (13.3)	56 113.9 (12.1)	0.027
Diabetes drug in baseline				
Biguanides	282 211.0 (58.2)	49 671.8 (57.9)	274 640.0 (59.0)	0.074
Thiazolidinediones	18 373.8 (3.8)	3164.1 (3.7)	17 551.9 (3.8)	0.072
Alpha‐glucosidase inhibitors	27 494.5 (5.7)	4638.8 (5.4)	25 857.5 (5.6)	0.021
Meglitinides	31 282.0 (6.5)	5721.0 (6.7)	29 458.5 (6.3)	0.021
SGLT2i	12 372.8 (2.6)	2053.8 (2.4)	11 394.3 (2.5)	0.020
GLP‐1 RA^c^	772.0 (0.2)	138.8 (0.2)	897.6 (0.2)	0.020
Insulin	70 011.5 (14.5)	12 035.9 (14.0)	60 959.0 (13.1)	0.020
Health care utilization, No. (%)				
Hospitalizations				
0	383 484.0 (79.1)	67 936.1 (79.2)	373 541 (80.3)	0.018
1	66 896.9 (13.8)	11 883.5 (13.9)	62 879.6 (13.5)	0.045
≥2	34 197.2 (7.1)	5910.1 (6.9)	28 975.4 (6.2)	0.028
Emergency department visits				
0	340 545.0 (70.3)	60 472.9 (70.5)	331 452.0 (71.2)	0.017
1	88 821.3 (18.3)	15 593 (18.2)	85 112.7 (18.3)	0.033
≥2	55211.8 (11.4)	9663.8 (11.3)	48831.6 (10.5)	0.021
HbA1C tests ordered				
0	89 619.8 (18.5)	15 569.9 (18.2)	86 150.6 (18.5)	0.013
1	129 628.0 (26.8)	22 807.9 (26.6)	125 791 (27.0)	0.048
≥2	265 331.0 (54.8)	47 351.9 (55.2)	253 454 (54.5)	0.012
Comorbidities, No. (%)				
Cardiovascular disease				
Myocardial infraction				
None	479 013.0 (98.9)	84 928.9 (99.1)	460 990.0 (99.1)	0.014
History	3224.1 (0.7)	431.5 (0.5)	2364.4 (0.5)	0.010
Hospitalisation	2341.3 (0.5)	369.3 (0.4)	2040.9 (0.4)	0.034
Ischemic stroke				
None	468 531.0 (96.7)	82 942.8 (96.8)	450 895.0 (96.9)	0.021
History	13 332.5 (2.8)	2335 (2.7)	12 026.9 (2.6)	0.009
Hospitalisation	2714.5 (0.6)	451.9 (0.5)	2473.7 (0.5)	0.011
Heart failure				
None	458 746.0 (94.7)	81 359.1 (94.9)	443 532.0 (95.3)	0.010
History	18 005.7 (3.7)	3103.5 (3.6)	16 104.7 (3.5)	0.009
Hospitalisation	7826.5 (1.6)	1267.1 (1.5)	5759.4 (1.2)	0.029
Cardiac arrhythmia				
None	455 983.0 (94.1)	80 723.2 (94.2)	439 859.0 (94.5)	0.032
History	23 309.1 (4.8)	4133.2 (4.8)	21 241.1 (4.6)	0.020
Hospitalisation	5286 (1.1)	873.3 (1.0)	4296.2 (0.9)	0.018
Haemorrhagic stroke	6752.2 (1.4)	1054.5 (1.2)	5576.8 (1.2)	0.012
Other stroke	21 580.2 (4.5)	3782.7 (4.4)	19 599.6 (4.2)	0.017
Ischemic heart disease	65 654.6 (13.6)	11 698.7 (13.7)	60 902.4 (13.1)	0.017
Coronary revascularization	3307.6 (0.7)	535.4 (0.6)	2740.6 (0.6)	0.012
Hypertension	268 680.0 (55.5)	47 762.1 (55.7)	255 212.0 (54.8)	0.014
Dyslipidaemia	225 552.0 (46.6)	40 142.9 (46.8)	219 048.0 (47.1)	0.012
Peripheral arterial disease	9729.4 (2.0)	1704.7 (2.0)	8750.6 (1.9)	0.012
Venous thromboembolism	2590.4 (0.5)	470.6 (0.6)	2364.6 (0.5)	0.010
Anaemia	19 628.9 (4.1)	3532.8 (4.1)	17 739.9 (3.8)	0.005
Thyroid disease	20 998.9 (4.3)	3690 (4.3)	19 816.3 (4.3)	0.005
Liver disease	57 709.7 (11.9)	10 159.2 (11.9)	56 412.9 (12.1)	0.012
Chronic kidney disease	84 727.6 (17.5)	15 228.3 (17.8)	75 994.5 (16.3)	0.005
Hyperkalaemia	2792.3 (0.6)	369.2 (0.4)	1792.9 (0.4)	0.007
Hypokalaemia	4824.7 (1.0)	787.3 (0.9)	3973.7 (0.9)	0.031
Cancer	18 571.7 (3.8)	3228.8 (3.8)	17 171 (3.7)	0.015
GERD	35 235.2 (7.3)	6376.3 (7.4)	33 635.9 (7.2)	0.007
Co‐medication, No. (%)				
Cardiovascular comedication				
ACEIs	29 303.0 (6.1)	5269.1 (6.2)	27 628.5 (5.9)	0.008
ARBs	182 690.0 (37.7)	32 897.4 (38.4)	173 410.0 (37.3)	0.003
Alpha‐blockers	3640.8 (0.8)	612.1 (0.7)	3387.2 (0.7)	0.005
Beta‐blockers	132 844.0 (27.4)	23 515.2 (27.4)	123 476.0 (26.5)	0.009
Calcium channel blockers				
Dihydropyridines	184 395.0 (38.1)	32 869.3 (38.3)	173 645.0 (37.3)	0.003
Non‐dihydropyridines	17 645.9 (3.6)	2913.8 (3.4)	15 828.5 (3.4)	0.020
Diuretics				
Thiazides	71 936.4 (14.9)	13 173.6 (15.4)	68 722.3 (14.8)	0.015
Loop	44 946.0 (9.3)	7851.5 (9.2)	37 764.5 (8.1)	0.013
Potassium‐sparing agents	15 849.6 (3.3)	2913.1 (3.4)	13 993.9 (3.0)	0.003
Antiplatelets	118611.0 (24.5)	20942.9 (24.4)	108748.0 (23.4)	0.041
Anticoagulants	21 151.0 (4.4)	3471.3 (4.1)	16 988.9 (3.7)	0.015
Statins	183 448.0 (37.9)	33 268.9 (38.8)	176 487.0 (37.9)	0.026
Others lipid‐lowering agents	38 227.1 (7.9)	6856.9 (8.0)	37 292.7 (8.0)	0.036
Nitrates	36 029.7 (7.4)	6059.2 (7.1)	31 357.5 (6.7)	0.002
Antiarrhythmics	16 352.0 (3.4)	2832.0 (3.3)	13 865.4 (3.0)	0.005
Digoxin	6458.3 (1.3)	1161.5 (1.4)	5833.4 (1.3)	0.027
Erythropoietin stimulating agents	11 243.7 (2.3)	1623.3 (1.9)	7176.0 (1.5)	0.023
Anti‐inflammatory agents				
NSAIDs	264 951.0 (54.7)	47 277.0 (55.2)	255 332.0 (54.9)	0.007
Steroids	101 084.0 (20.9)	18 046.4 (21.1)	94 835.5 (20.4)	0.057
K channel opener	10 013.3 (2.1)	1670.1 (2.0)	8872.9 (1.9)	0.004
Inhibitors of mitochondrial PT pore	13 0176.0 (26.9)	23 049.3 (26.9)	120 874.0 (26.0)	0.012
Proton pump inhibitors	42 344.6 (8.7)	7294.4 (8.5)	37 545.7 (8.1)	0.012

Abbreviations: ACEIs, angiotensin converting enzyme inhibitors; aDSCI, adapted Diabetes Complications Severity Index; ARBs, angiotensin receptor blockers; aSMD, absolute standardised mean difference; DM, diabetes mellitus; DPP‐4 inhibitors, dipeptidyl peptidase‐4 inhibitors; GERD, gastroesophageal reflux disease; HbA1c, glycated haemoglobin; MitoK_ATP_, mitochondrial ATP‐sensitive potassium channel; No., number; NSAIDs, nonsteroidal anti‐inflammatory drugs; NTD, New Taiwan dollar; PT, permeability transition; SGLT2i, sodium‐glucose co‐transporter 2 inhibitor; SD, standard deviation.

^a^ All co‐medications, diabetes severity indicators, health care utilisation, and monthly income were measured in the year preceding the cohort entry date; all comorbidities and diabetes drugs at baseline were measured 180 days preceding the cohort entry date; age, sex, entry year, and hospital level were measured at the cohort entry date.

^b^ Standardized mean difference >0.1 represents meaningful differences between two groups.

^c^ GLP‐1RA was not included in the PS model because the concomitant use with DPP‐4 inhibitors is not recommended based on the reimbursement policy of the National Health Insurance program.

Cox proportional hazard models were employed to estimate hazard ratios (HRs) and 95% confidence intervals (CIs) of all examined outcomes using IPTW and robust variance estimation. The proportional hazards assumption was assessed by adding an interaction term between exposure status and log(time). No significant violations of the assumption were found. Kaplan–Meier plots were also used to provide the accumulative incidence rate of each examined outcome for all three treatments of interest during follow‐up time.

## 3 RESULTS

A total of 1 161 254 adults with type 2 diabetes mellitus initiating therapy with low‐affinity cardiac mitoK_ATP_ channel sulfonylureas, high‐affinity sulfonylureas, and DPP‐4 inhibitors were identified. After applying the exclusion criteria, the study cohort included 466 158 patients initiating low‐affinity sulfonylureas, 83 031 new users of high‐affinity sulfonylureas, and 473 539 initiators of DPP‐4 inhibitors (Figure S1). The study cohort's mean (SD) age was 60.22 (13.80) years, with 55.6% being male. The weighted mean (SD) follow‐up duration across the groups was 20.37 (22.97) months, with reasons for truncation provided in Table S3.

Baseline patient characteristics, as defined in the covariate measurement section, across the three groups prior to IPTW are detailed in Table S4. Compared with DPP4‐inhibitor initiators, patients starting low‐affinity and high‐affinity sulfonylureas had generally comparable baseline characteristics but were more often enrolled in the first two calendar years of the study, had lower aDSCI scores, less often received insulin, or had a slightly lower prevalence of certain cardiovascular disease histories (the differences <3%), including MI and heart failure. Following IPTW, covariate balance across the groups was well achieved (Table 1 and S5).

Table 2 presents the incidence rates per 1000 person‐years of outcomes across the three treatment groups. The weighted incidence rates of 3‐point MACE were 13.05, 13.82, and 17.99 per 1000 person‐years for DPP‐4 inhibitors, low‐affinity sulfonylureas, and high‐affinity sulfonylureas, respectively. Patients receiving high‐affinity cardiac mitoK_ATP_ channel sulfonylureas exhibited the highest incidence rates across all components of MACE and secondary outcomes, while those on DPP‐4 inhibitors had the lowest. Except for hypoglycaemia, the incidence rates of primary and secondary outcomes were comparable between the low‐affinity mitoK_ATP_ channel sulfonylurea group and DPP‐4 inhibitor group. Cumulative incidence rates for each outcome among the three groups, estimated using the Kaplan–Meier method, are shown in Figures S2 and S3.

**TABLE 2:** Number of events and incidence rates per 1000 person‐years of primary and secondary outcomes among patients receiving MitoK_ATP_ low‐affinity SU, MitoK_ATP_ high‐affinity SU, and DPP‐4 inhibitors.

	MitoK_ATP_ low‐affinity SU (*n* = 466 158)			MitoK_ATP_ high‐affinity SU (*n* = 83 031)			DPP‐4 inhibitors (*n* = 473 539)		
	Events	Incidence rate (1000 person‐years)		Events	Incidence rate (1000 person‐years)		Events	Incidence rate (1000 person‐years)	
		Crude	Weighted		Crude	Weighted		Crude	Weighted
Primary outcome									
3‐point MACE	7880	10.75	13.82	1939	17.37	17.99	12 696	14.92	13.05
Secondary outcomes									
Myocardial infarction	1830	2.48	2.93	377	3.36	3.39	2658	3.10	2.66
Ischemic stroke	4298	5.85	6.42	971	8.68	7.97	5151	6.03	5.80
CV death	4654	6.30	11.86	1406	12.51	14.87	12 202	14.20	11.15
Heart failure	1855	2.52	4.16	442	3.94	4.51	4275	5.00	3.98
Cardiac arrhythmia	656	0.89	1.24	153	1.36	1.53	1270	1.48	1.26
Hypoglycaemia	1079	1.46	1.85	354	3.15	2.80	660	0.77	0.65
All‐cause mortality	11 274	15.27	27.15	3768	33.55	39.89	28 269	32.92	26.40

Abbreviations: 3‐point MACE, 3‐point major cardiovascular events outcome; CV, cardiovascular; DPP‐4 inhibitors, dipeptidyl peptidase 4 inhibitors; MitoK_ATP_, mitochondrial ATP‐sensitive potassium channel; SU, sulfonylurea.

Compared to DPP‐4 inhibitors, low‐affinity cardiac mitoK_ATP_ channel sulfonylureas were not associated with an increased risk of 3‐point MACE (weighted HR, 1.01; 95% CI, 0.96–1.06) or its individual components (Table 3), but were associated with a 2.58‐fold (95% CI, 2.24–2.98) higher risk of hypoglycaemia. In contrast, high‐affinity sulfonylureas were associated with a 1.23‐fold increased risk of 3‐point MACE (95% CI, 1.14–1.33), corresponding to an *E*‐value of 1.76 (lower bound, 1.54), a 1.19‐fold (95% CI, 1.01–1.41) risk of MI, a 1.23‐fold risk of ischemic stroke (95% CI, 1.12–1.34), and a 1.17‐fold risk of cardiovascular death (95% CI, 1.05–1.30). Additionally, use of high‐affinity sulfonylureas was associated with a 3.48‐fold (95% CI, 2.67–4.55) increased risk of hypoglycaemia and a 31% (HR, 1.31; 95% CI, 1.22–1.40) increased risk of all‐cause mortality compared to DPP‐4 inhibitors. Furthermore, low‐affinity sulfonylureas were associated with a lower MACE risk than high‐affinity agents (weighted HR, 0.82; 95% CI, 0.75–0.89; Table 4). The number needed to harm for each outcome in the pairwise comparisons was presented in Table S6.

**TABLE 3:** Comparative risk of primary and secondary outcomes among MitoK_ATP_ low‐affinity SU, MitoK_ATP_ high‐affinity SU, and DPP‐4 inhibitors.

	MitoK_ATP_ low‐affinity SU vs. DPP‐4 inhibitors		MitoK_ATP_ high‐affinity SU vs. DPP‐4 inhibitors	
	HR (95% CI)		HR (95% CI)	
	Crude	Weighted	Crude	Weighted
Primary outcomes					
3‐point MACE	0.71 (0.69–0.73)*	1.01 (0.96–1.06)	1.08 (1.03–1.13)*	1.23 (1.14–1.33)*
Secondary outcomes					
Myocardial infarction	0.80 (0.75–0.84)*	1.07 (0.98–1.17)	1.04 (0.93–1.16)	1.19 (1.01–1.41)*
Ischemic stroke	0.96 (0.92–0.99)*	1.05 (0.99–1.11)	1.33 (1.24–1.42)*	1.23 (1.12–1.34)*
CV death	0.44 (0.42–0.45)*	1.01 (0.93–1.08)	0.81 (0.77–0.86)*	1.17 (1.05–1.30)*
Heart failure	0.50 (0.47–0.53)*	0.99 (0.84–1.17)	0.73 (0.66–0.80)*	1.00 (0.86–1.15)
Cardiac arrhythmia	0.60 (0.54–0.66)*	0.96 (0.84–1.09)	0.88 (0.74–1.04)	1.13 (0.82–1.56)
Hypoglycaemia	1.86 (1.69–2.05)*	2.58 (2.24–2.98)*	3.51 (3.08–3.99)*	3.48 (2.67–4.55)*
All‐cause mortality	0.454 (0.445–0.47)	0.97 (0.93–1.01)	0.93 (0.90–0.96)	1.31 (1.22–1.40)*

Abbreviations: 3‐point MACE, 3‐point major cardiovascular events outcome; CI, confidence interval; CV, cardiovascular; DPP‐4 inhibitors, dipeptidyl peptidase 4 inhibitors; HR, hazard ratio; MitoK_ATP_, mitochondrial ATP‐sensitive potassium channel; SU, sulfonylurea; vs., versus.

* *p* < 0.05.

**TABLE 4:** Comparative risk of major adverse cardiovascular events between MitoK_ATP_ low‐affinity SU and MitoK_ATP_ high‐affinity SU.

	MitoK_ATP_ low‐affinity SU	MitoK_ATP_ high‐affinity SU	MitoK_ATP_ low‐affinity SU versus MitoK_ATP_ high‐affinity SU	
	*N* = 466 158	*N* = 83 031	HR (95% CI)	
	N events (IR/1000 PY)	N events (IR/1000 PY)	Crude	Weighted
Primary outcome				
3‐point MACE	7880 (13.82)	1939 (17.99)	0.66 (0.63–0.69)*	0.82 (0.75–0.89)*
Secondary outcomes				
MI	1830 (2.93)	377 (3.39)	0.77 (0.69–0.86)*	0.90 (0.75–1.08)
Ischemic stroke	4298 (6.42)	971 (7.97)	0.72 (0.67–0.77)*	0.86 (0.78–0.94)*
CV death	4654 (11.86)	1406 (14.87)	0.54 (0.51–0.57)*	0.86 (0.75–0.98)*
HF	1855 (4.16)	442 (4.51)	0.68 (0.62–0.76)*	0.99 (0.79–1.24)
Arrhythmia	656 (1.24)	153 (1.53)	0.68 (0.57–0.81)	0.85 (0.61–1.18)
Hypoglycaemia	1079 (1.85)	354 (2.80)	0.53 (0.47–0.60)*	0.74 (0.56–0.98)*
All‐cause mortality	11 274 (27.15)	3768 (39.89)	0.49 (0.47–0.51)*	0.74 (0.68–0.80)*

Abbreviations: 3‐point MACE, 3‐point major cardiovascular events outcome; CI, confidence interval; CV, cardiovascular; HF, heart failure; HR, hazard ratio; IR/1000 PY, incident rate/1000 person‐years; MI, myocardial infarction; MitoK_ATP_, mitochondrial ATP‐sensitive potassium channel; SU, sulfonylurea.

* *p* < 0.05.

**FIGURE 1:**
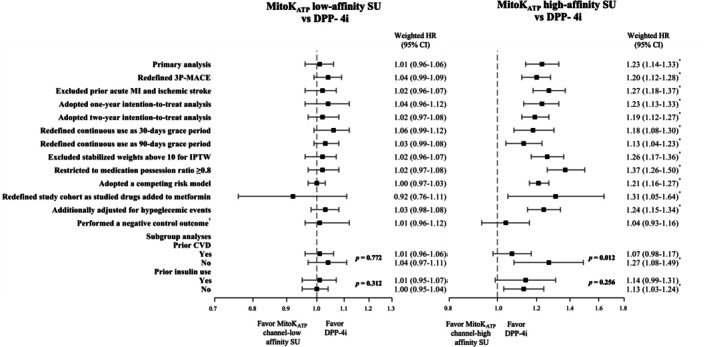
Forest plots showing the sensitivity and subgroup analyses of the primary analysis. aHR, adjusted hazard ratio; MACEs, major adverse cardiovascular events; MitoK_ATP_, mitochondrial adenosine triphosphate sensitive‐potassium. *p < 0.05. *p*‐values in subgroup analyses represent the interaction analyses.

The main results persisted across all sensitivity analyses comparing low‐ and high‐affinity cardiac mitoK_ATP_ channel sulfonylureas versus DPP‐4 inhibitors, respectively (Figure 1). These analyses included intention‐to‐treat analyses, redefinition of continuous use, and restricting the study cohort to patients who initiated metformin and subsequently added sulfonylureas or DPP‐4 inhibitors. No associations were observed for the negative control outcome. Subgroup analyses indicated that prior cardiovascular disease modified the effect of high‐affinity sulfonylureas versus DPP‐4 inhibitors (*P* for interaction = 0.012). Among patients with prior cardiovascular disease, the increased 3‐point MACE risk associated with high‐affinity sulfonylureas was attenuated and not statistically significant compared with DPP‐4 inhibitors (HR, 1.07; 95% CI, 0.98–1.17).

## 4 DISCUSSION

In this large nationwide cohort study of 1 022 728 type 2 diabetes patients treated with one of three antidiabetic medications, we found that, compared with DPP‐4 inhibitors, (1) initiating treatment with low‐affinity cardiac mitoK_ATP_ channel sulfonylureas was not associated with an increased MACE risk; however, (2) starting therapy with high‐affinity cardiac mitoK_ATP_ channel sulfonylureas was associated with a 1.23‐fold increased MACE risk. Given the generally neutral cardiovascular profile of DPP‐4 inhibitors, our findings suggest that high‐affinity sulfonylureas may increase the risk of major cardiovascular events, whereas low‐affinity sulfonylureas do not. The primary findings were robust across the sensitivity analyses.

The cardiovascular safety of sulfonylureas has remained controversial for nearly five decades. Cardiovascular outcomes trials for older agents, such as sulfonylureas, are limited; previous observational studies have yielded contradictory findings, primarily due to selection bias,^15^, ^16^ time‐lag bias,^12^, ^13^, ^14^ and comparison with metformin^7^, ^8^, ^9^, ^20^ (an agent with debated cardiovascular benefits), and lack of rigorous study design,^6^ with most prior studies assessing sulfonylureas as a whole class rather than individual agents. Our prior research suggests that blockade of cardiac mitoK_ATP_ channels is a key mechanism underlying sulfonylurea‐associated cardiovascular risk, as high‐affinity sulfonylureas are associated with increased risk of MACE compared with low‐affinity agents when used as initial monotherapy or in combination with metformin in diabetic patients.^22^, ^23^ Expanding on these results, this investigation examined a more diverse cohort with longer disease duration, higher aDCSI scores, and greater cardiovascular comorbidity. In this population, low‐affinity sulfonylureas were consistently associated with lower MACE risk than high‐affinity sulfonylureas (Table S6). Most importantly, addressing these limitations, our current study compared high‐affinity sulfonylureas and low‐affinity sulfonylureas with DPP‐4 inhibitors (which generally have a neutral cardiovascular effect). These findings suggest that high‐affinity sulfonylureas may confer a higher risk of cardiovascular events, whereas low‐affinity sulfonylureas may have a cardiovascular safety profile similar to DPP‐4 inhibitors.

Two large randomised controlled trials and one observational study have reported neutral cardiovascular risk for certain low‐affinity mitoK_ATP_ channel sulfonylureas.^17^, ^38^ The Cardiovascular Outcome Study of Linagliptin versus Glimepiride in Type 2 Diabetes (CAROLINA) found no significant difference (HR, 0.98; 95.47% CI, 0.84–1.14) in 3‐point MACE risk between linagliptin, a DPP‐4 inhibitor, and glimepiride among patients with type 2 diabetes at high cardiovascular risk.^38^ Similarly, the Thiazolidinediones or Sulfonylureas Cardiovascular Accidents Intervention Trial (TOSCA.IT) reported no significant difference in cardiovascular outcomes (HR, 0.96; 95% CI, 0.74–1.26) between thiazolidinedione, pioglitazone, and sulfonylureas,^17^ of which 98% were low‐affinity sulfonylurea users (gliclazide and glimepiride) among individuals with type 2 diabetes largely free of prior cardiovascular events and inadequately controlled with metformin. Additionally, this clinical trial adopted pioglitazone as the comparator, which has been documented to have a beneficial MACE effect,^39^ although such effect could be confined to patients with prior cardiovascular diseases. Another Scottish observational study found no difference in MACE risk between sulfonylureas and DPP‐4 inhibitors/thiazolidinedione (HR, 1.00; 95% CI: 0.91–1.09), with 87% of sulfonylureas use involving gliclazide.^40^ Accordingly, such findings may not generalise to all sulfonylureas with differing affinities for mitoK_ATP_ channels. Although a stratified analysis by individual sulfonylurea revealed no variation in MACE risk, this finding might be attributable to insufficient statistical power due to the small sample size in the subgroup of high‐affinity sulfonylureas.

Our observed increased risks of MACE and cardiovascular deaths associated with high‐affinity sulfonylureas may result from inhibition of cardiac mitoK_ATP_ channels, which are essential for ischaemic preconditioning and myocardial adaptation to oxygen deprivation.^41^ This mechanism likely contributes to the elevated all‐cause mortality, given the substantial role of cardiovascular death in type 2 diabetes. However, the higher HR for all‐cause mortality compared with cardiovascular death suggests that non‐cardiovascular causes may also contribute. Conversely, the minimal mitoK_ATP_ blockade of low‐affinity sulfonylureas may explain their neutral cardiovascular profile. The absence of excess risk from high‐affinity sulfonylureas in patients with prior cardiovascular disease, despite the potentially limited statistical power of this subgroup analysis, may reflect loss of ischaemic preconditioning from repeated ischaemia or infarction, where mitoK_ATP_‐mediated protection may already be compromised and further inhibition exerts minimal additional impact.^42^, ^43^

Current study offers several strengths. It is the first to evaluate the cardiovascular safety of sulfonylureas by their differential affinities for cardiac mitoK_ATP_ channels using a cardiovascular‐neutral comparator. Parsimonious exclusion criteria yielded a diverse, nationwide type 2 diabetes cohort, enhancing generalisability to similar real‐world clinical settings, though limited to an Asian population and without adjustment for multiple testing. Primary outcomes were robustly ascertained using validated coding algorithms for MI and ischaemic stroke, with cardiovascular deaths determined from the national death registry. The large sample size provided adequate power for the primary outcome, and low, non‐differential out‐of‐pocket costs across antidiabetic classes under Taiwan's NHI likely minimised socioeconomic confounding.

Our study has several potential limitations. First, unmeasured confounding is inherent in observational studies, partly due to unavailable laboratory values (e.g., HbA1c). However, we minimised this risk using an active‐comparator, PS‐IPTW design and further assessed it through a negative outcome analysis. E‐value analyses demonstrate confounding is unlikely to fully explain our findings, though high‐dimensional PS approaches could provide further reassurance. Second, substantial differences in the follow‐up duration and truncation reasons for some outcomes may introduce informative censoring bias. Nevertheless, in our 1‐ and 2‐year intention‐to‐treat analyses, follow‐up times were similar across the groups (data not shown), and the results were consistent with the main findings. Third, medication adherence may have confounded the results. Although restricting to patients with MPR ≥0.8 addresses primary nonadherence, secondary nonadherence remains possible, as patients may not take medications after refilling. Fourth, time‐lag bias may arise when comparing sulfonylureas with DPP‐4 inhibitors used at different diabetic stages. We conducted a sensitivity analysis restricted to metformin monotherapy initiators, comparing add‐on sulfonylureas with add‐on DPP‐4 inhibitors and adjusting for metformin duration. Results were consistent with the primary findings, suggesting minimal impact of this bias, though advanced approaches (e.g., Fox & Weisberg's model)^44^ could further support. Lastly, before weighting, baseline characteristics differed substantially across groups, and for some outcomes, HRs changed after IPTW. The mean weight was 1.01, and excluding extreme weights (≥10) yielded results consistent with the main findings.

## 5 CONCLUSIONS

This nationwide cohort study found that low‐affinity mitoK_ATP_ channel sulfonylureas were not associated with increased 3‐point MACE risks, cardiovascular deaths, and all‐cause mortality compared to DPP‐4 inhibitors. However, high‐affinity sulfonylureas were linked to significantly increased risks across these outcomes when compared to DPP‐4 inhibitors. These findings suggest that low‐affinity sulfonylureas are preferable to high‐affinity agents and are as safe as DPP‐4 inhibitors. They may be considered appropriate options for patients with limited access to, or who do not require, newer antidiabetic therapies.

## AUTHOR CONTRIBUTIONS

All authors conceptualised and designed the current study; Wang MT acquired data from the database; Wang MT and Hung TY analysed the data; Chen MH, Lin LY, Hung TY, Li CY, Lin TH, Chen YH, and Liou JT interpreted the data; Chen MH, Lin TC, and Wang MT drafted the manuscript; all authors critically revised and approved the submitted manuscript. Wang MT is the guarantor of this work and, as such, had full access to all the data in the study and takes responsibility for the integrity of the data and the accuracy of the data analysis.

## FUNDING INFORMATION

This study was funded by Far Eastern Memorial Hospital National Yang Ming Chiao Tung University Joint Research Program (#NYCU‐FEMH113DN01), and partially supported by the Far Eastern Memorial Hospital (#FEMH‐2024‐C‐057).

## CONFLICT OF INTEREST STATEMENT

The authors declare no conflict of interest.

## PEER REVIEW

The peer review history for this article is available at https://www.webofscience.com/api/gateway/wos/peer‐review/10.1111/dom.70157.

## ETHICS STATEMENT

Ethics of the study has been approved by the Institutional Review Board of the National Yang Ming Chiao Tung University (NYCU112142AE) and Taipei Veterans General Hospital (2023‐08‐013BC), and a waiver of informed consent has been granted because of the analysis of de‐identified health care records.

## Supporting information


**Appendix S1:** Supporting information.

## Data Availability

According to the regulations of Taiwan's Ministry of Health and Welfare (https://dep.mohw.gov.tw/DOS/cp-5119-59201-113.html), the analysed raw data from this study cannot be shared publicly. To request access to the analysed data, please contact the Health and Welfare Data Science Center, Ministry of Health and Welfare, Taiwan (https://dep.mohw.gov.tw/DOS/cp-5119-59201-113.html).
