# Supplementary material for: Comparative cardiovascular risk of sulfonylureas with low‐ and high‐affinities for cardiac mitochondrial adenosine triphosphate‐sensitive potassium channels versus dipeptidyl peptidase‐4 inhibitors in patients with type 2 diabetes: A cohort study
Source: Diabetes Obes Metab. 2025 Sep 29;27(12):7502–13. doi: 10.1111/dom.70157 (PMC12587235; doi:10.1111/dom.70157)
Supplement: Supplementary file 1 — Appendix S1: Supporting information. [file DOM-27-7502-s001.docx]

**Supplementary materials to “Comparative Cardiovascular Risk of Sulfonylureas with Low- and High- Affinities for Cardiac Mitochondrial Adenosine Triphosphate–Sensitive Potassium Channels versus Dipeptidyl Peptidase-4 Inhibitors in Patients with Type 2 Diabetes: A Cohort Study”**

Table of Contents

[e-Table 1. Operational definitions for the adopted exclusion criteria, safety outcomes, comorbidities, and co-medications 2](#_Toc207700726)

[e-Table-2. Sensitivity analysis and their purposes 9](#_Toc207700727)

[e-Table 3. The mean duration and reasons for truncation during follow-up within each group by outcomes 11](#_Toc207700728)

[e-Table 3. The mean duration and reasons for truncation during follow-up within each group by outcomes (continued) 12](#_Toc207700729)

[e-Table 3. The mean duration and reasons for truncation during follow-up within each group by outcomes (continued) 13](#_Toc207700730)

[e-Table 4. Demographic and clinical characteristics of users of MitoK_ATP_ channel-low affinity sulfonylureas, MitoK_ATP_ channel-high affinity sulfonylureas, and DPP-4 inhibitors before propensity score-based IPTW 14](#_Toc207700731)

[e-Table 5. Demographic and clinical characteristics of users of MitoK_ATP_ channel-low affinity sulfonylureas, MitoK_ATP_ channel-high affinity sulfonylureas, and DPP-4 inhibitors after propensity score-based IPTW (Full version) 19](#_Toc207700732)

[e-Table 6. The patient-based number needed to harm for the comparative results across three groups 25](#_Toc207700733)

[e-Figure 1. Study flow diagram illustrating the selection of users of MitoK_ATP_ channel-low affinity sulfonylureas, MitoK_ATP_ channel-high affinity sulfonylureas, and DPP-4 inhibitors 26](#_Toc207700734)

[e-Figure 2. Kaplan-Meier survival curves of 3-point MACE (A), MI (B), Ischemic stroke (C), and CV death (D) among three groups 27](#_Toc207700735)

[e-Figure 3. Kaplan-Meier survival curves of HF (A), Arrhythmia (B), Hypoglycemia (C), and All-cause mortality (D) among three groups 28](#_Toc207700736)

[e-Methods 1. Analysis of patients with metformin monotherapy before adding sulfonylureas or DPP-4 inhibitors. 29](#_Toc207700737)

# e-Table 1. Operational definitions for the adopted exclusion criteria, safety outcomes, comorbidities, and co-medications

| **Exclusion criteria** | **Diagnosis codes** |
| --- | --- |
| Type 1 DM diagnosis | ICD-9 250.x1 250.x3；ICD-10 E10 |
| Pregnancy | ICD-9 630-677; ICD-10 O00-O99, O9A |
| Hospitalization for |  |
| Acute MI | ICD-9 410; ICD-10 I21 |
| Coronary revascularization | ICD-9-CM procedure code: 00.66, 17.55, 36.0, 36.1, 36.2; ICD-10-PCS code: 0210093, 0210098, 0210099, 021009C, 021009F, 021009W, 02100A3, 02100A8, 02100A9, 02100AC, 02100AF, 02100AW, 02100J3, 02100J8, 02100J9, 02100JC, 02100JF, 02100JW, 02100K3, 02100K8, 02100K9, 02100KC, 02100KF, 02100KW, 02100Z3, 02100Z8, 02100Z9, 02100ZC, 02100ZF, 0210493, 0210498, 0210499, 021049C, 021049F, 021049W, 02104A3, 02104A8, 02104A9, 02104AC, 02104AF, 02104AW, 02104J3, 02104J8, 02104J9, 02104JC, 02104JF, 02104JW, 02104K3, 02104K8, 02104K9, 02104KC, 02104KF, 02104KW, 02104Z3, 02104Z8, 02104Z9, 02104ZC, 02104ZF, 0211093, 0211098, 0211099, 021109C, 021109F, 021109W, 02110A3, 02110A8, 02110A9, 02110AC, 02110AF, 02110AW, 02110J3, 02110J8, 02110J9, 02110JC, 02110JF, 02110JW, 02110K3, 02110K8, 02110K9, 02110KC, 02110KF, 02110KW, 02110Z3, 02110Z8, 02110Z9, 02110ZC, 02110ZF, 0211493, 0211498, 0211499, 021149C, 021149F, 021149W, 02114A3, 02114A8, 02114A9, 02114AC, 02114AF, 02114AW, 02114J3, 02114J8, 02114J9, 02114JC, 02114JF, 02114JW, 02114K3, 02114K8, 02114K9, 02114KC, 02114KF, 02114KW, 02114Z3, 02114Z8, 02114Z9, 02114ZC, 02114ZF, 0212093, 0212098, 0212099, 021209C, 021209F, 021209W, 02120A3, 02120A8, 02120A9, 02120AC, 02120AF, 02120AW, 02120J3, 02120J8, 02120J9, 02120JC, 02120JF, 02120JW, 02120K3, 02120K8, 02120K9, 02120KC, 02120KF, 02120KW, 02120Z3, 02120Z8, 02120Z9, 02120ZC, 02120ZF, 0212493, 0212498, 0212499, 021249C, 021249F, 021249W, 02124A3, 02124A8, 02124A9, 02124AC, 02124AF, 02124AW, 02124J3, 02124J8, 02124J9, 02124JC, 02124JF, 02124JW, 02124K3, 02124K8, 02124K9, 02124KC, 02124KF, 02124KW, 02124Z3, 02124Z8, 02124Z9, 02124ZC, 02124ZF, 0213093, 0213098, 0213099, 021309C, 021309F, 021309W, 02130A3, 02130A8, 02130A9, 02130AC, 02130AF, 02130AW, 02130J3, 02130J8, 02130J9, 02130JC, 02130JF, 02130JW, 02130K3, 02130K8, 02130K9, 02130KC, 02130KF, 02130KW, 02130Z3, 02130Z8, 02130Z9, 02130ZC, 02130ZF, 0213493, 0213498, 0213499, 021349C, 021349F, 021349W, 02134A3, 02134A8, 02134A9, 02134AC, 02134AF, 02134AW, 02134J3, 02134J8, 02134J9, 02134JC, 02134JF, 02134JW, 02134K3, 02134K8, 02134K9, 02134KC, 02134KF, 02134KW, 02134Z3, 02134Z8, 02134Z9, 02134ZC, 02134ZF, 021K0Z8, 021K0Z9, 021K0ZC, 021K0ZF, 021K0ZW, 021K4Z8, 021K4Z9, 021K4ZC, 021K4ZF, 021K4ZW, 021L09P, 021L09Q, 021L09R, 021L0AP, 021L0AQ, 021L0AR, 021L0JP, 021L0JQ, 021L0JR, 021L0KP, 021L0KQ, 021L0KR, 021L0Z8, 021L0Z9, 021L0ZC, 021L0ZF, 021L0ZP, 021L0ZQ, 021L0ZR, 021L49P, 021L49Q, 021L49R, 021L4AP, 021L4AQ, 021L4AR, 021L4JP, 021L4JQ, 021L4JR, 021L4KP, 021L4KQ, 021L4KR, 021L4Z8, 021L4Z9, 021L4ZC, 021L4ZF, 021L4ZP, 021L4ZQ, 021L4ZR, 02700ZZ, 02703ZZ, 02704ZZ, 02710ZZ, 02713ZZ, 02714ZZ, 02720ZZ, 02723ZZ, 02724ZZ, 02730ZZ, 02733ZZ, 02734ZZ, 02C00ZZ, 02C03ZZ, 02C04ZZ, 02C10ZZ, 02C13ZZ, 02C14ZZ, 02C20ZZ, 02C23ZZ, 02C24ZZ, 02C30ZZ, 02C33ZZ, 02C34ZZ, 3E07017, 3E070PZ, 3E07317, 3E073PZ; NHI procedure code: 68023B, 68024B, 68025B, 68053B, 68054B, 68055B, 3076B, 33077B, 33078B, N26002, N26003 |
| Unstable angina | ICD-9 411.1; ICD-10 I20.0, I24.xx, I25.110, 125.7x0 |
| Ischemic or hemorrhagic stroke | ICD-9 433 and 434; ICD-10 I63-I66  ICD 9: 430-432; ICD 10: I60-I61 |
| Transient ischemic attack | ICD-9 435; ICD-10 G45.8, G45.9 |
| Heart failure | ICD 9: 428; ICD 10: I11.0, I13.0, I13.2, I50 |
| **Primary outcomes definition** | **Diagnosis codes** |
| Myocardial infarction | ICD-9 410; ICD-10 I21 |
| Ischemic stroke | ICD-9 433 and 434; ICD-10 I63-I66 |
| CV death | ICD-10 I00-I99 (Linked with the National Death Registry records) |
| **Secondary outcomes definition** | **Diagnosis codes** |
| Myocardial infarction | ICD-9 410; ICD-10 I21 |
| Ischemic stroke | ICD-9 433 and 434; ICD-10 I63-I66 |
| CV death | ICD-10 I00-I99 (Linked with the National Death Registry records) |
| Arrhythmia | ICD-9 427; ICD 10 I47-49 |
| Heart failure | ICD-9 428；ICD-10 I11.0、I13.0、I13.2、I50 |
| Hypoglycemia | ICD-9 code: 251.0-2 and 250.8 (without 259.8, 272.7, 681, 682, 686.9, 707, 709.3, 730.0–730.2, and 731.8)  ICD-10: E08.641, E08.649, E09.641, E09.649, E11.641, E11.649, E13.641, E13.649, E15, E16.0, E16.1, E16.2, E11.63, E13.63, E14.63 |
| **Comorbidities** | **Diagnosis codes** |
| CV diseases |  |
| Myocardial infraction | ICD-9: 410; ICD-10: I21 |
| Ischemic Stroke | ICD 9: 433 and 434; ICD 10: I63-I66 |
| Heart failure | ICD-9: 428; ICD 10: I11.0, I13.0, I13.2, I50 |
| Cardiac arrhythmia | ICD 9: 427; ICD 10: I47-I49 |
| Hemorrhagic stroke | ICD-9: 430-432; ICD-10: I60-I61 |
| Other strokes | ICD-9: 435-438; ICD-10: I62, I67-I69, G45-46 |
| Ischemic heart disease | ICD 9: 411-414; ICD 10: I20, I22-I25 |
| Hypertension | ICD 9: 401-405; ICD 10: I10-I15 |
| Dyslipidemia | ICD 9: 272; ICD 10: E71.30, E75.21, E75.22, E75.24, E75.3, E75.5, E75.6, E77, E78, E88.1, E88.2, E88.89 |
| Peripheral arterial disease | ICD 9: 440, 443.9; ICD 10: I70, I75, I73.9 |
| Coronary revascularization | ICD-9-CM procedure code: 00.66, 17.55, 36.0, 36.1, 36.2; ICD-10-PCS code: 0210093, 0210098, 0210099, 021009C, 021009F, 021009W, 02100A3, 02100A8, 02100A9, 02100AC, 02100AF, 02100AW, 02100J3, 02100J8, 02100J9, 02100JC, 02100JF, 02100JW, 02100K3, 02100K8, 02100K9, 02100KC, 02100KF, 02100KW, 02100Z3, 02100Z8, 02100Z9, 02100ZC, 02100ZF, 0210493, 0210498, 0210499, 021049C, 021049F, 021049W, 02104A3, 02104A8, 02104A9, 02104AC, 02104AF, 02104AW, 02104J3, 02104J8, 02104J9, 02104JC, 02104JF, 02104JW, 02104K3, 02104K8, 02104K9, 02104KC, 02104KF, 02104KW, 02104Z3, 02104Z8, 02104Z9, 02104ZC, 02104ZF, 0211093, 0211098, 0211099, 021109C, 021109F, 021109W, 02110A3, 02110A8, 02110A9, 02110AC, 02110AF, 02110AW, 02110J3, 02110J8, 02110J9, 02110JC, 02110JF, 02110JW, 02110K3, 02110K8, 02110K9, 02110KC, 02110KF, 02110KW, 02110Z3, 02110Z8, 02110Z9, 02110ZC, 02110ZF, 0211493, 0211498, 0211499, 021149C, 021149F, 021149W, 02114A3, 02114A8, 02114A9, 02114AC, 02114AF, 02114AW, 02114J3, 02114J8, 02114J9, 02114JC, 02114JF, 02114JW, 02114K3, 02114K8, 02114K9, 02114KC, 02114KF, 02114KW, 02114Z3, 02114Z8, 02114Z9, 02114ZC, 02114ZF, 0212093, 0212098, 0212099, 021209C, 021209F, 021209W, 02120A3, 02120A8, 02120A9, 02120AC, 02120AF, 02120AW, 02120J3, 02120J8, 02120J9, 02120JC, 02120JF, 02120JW, 02120K3, 02120K8, 02120K9, 02120KC, 02120KF, 02120KW, 02120Z3, 02120Z8, 02120Z9, 02120ZC, 02120ZF, 0212493, 0212498, 0212499, 021249C, 021249F, 021249W, 02124A3, 02124A8, 02124A9, 02124AC, 02124AF, 02124AW, 02124J3, 02124J8, 02124J9, 02124JC, 02124JF, 02124JW, 02124K3, 02124K8, 02124K9, 02124KC, 02124KF, 02124KW, 02124Z3, 02124Z8, 02124Z9, 02124ZC, 02124ZF, 0213093, 0213098, 0213099, 021309C, 021309F, 021309W, 02130A3, 02130A8, 02130A9, 02130AC, 02130AF, 02130AW, 02130J3, 02130J8, 02130J9, 02130JC, 02130JF, 02130JW, 02130K3, 02130K8, 02130K9, 02130KC, 02130KF, 02130KW, 02130Z3, 02130Z8, 02130Z9, 02130ZC, 02130ZF, 0213493, 0213498, 0213499, 021349C, 021349F, 021349W, 02134A3, 02134A8, 02134A9, 02134AC, 02134AF, 02134AW, 02134J3, 02134J8, 02134J9, 02134JC, 02134JF, 02134JW, 02134K3, 02134K8, 02134K9, 02134KC, 02134KF, 02134KW, 02134Z3, 02134Z8, 02134Z9, 02134ZC, 02134ZF, 021K0Z8, 021K0Z9, 021K0ZC, 021K0ZF, 021K0ZW, 021K4Z8, 021K4Z9, 021K4ZC, 021K4ZF, 021K4ZW, 021L09P, 021L09Q, 021L09R, 021L0AP, 021L0AQ, 021L0AR, 021L0JP, 021L0JQ, 021L0JR, 021L0KP, 021L0KQ, 021L0KR, 021L0Z8, 021L0Z9, 021L0ZC, 021L0ZF, 021L0ZP, 021L0ZQ, 021L0ZR, 021L49P, 021L49Q, 021L49R, 021L4AP, 021L4AQ, 021L4AR, 021L4JP, 021L4JQ, 021L4JR, 021L4KP, 021L4KQ, 021L4KR, 021L4Z8, 021L4Z9, 021L4ZC, 021L4ZF, 021L4ZP, 021L4ZQ, 021L4ZR, 02700ZZ, 02703ZZ, 02704ZZ, 02710ZZ, 02713ZZ, 02714ZZ, 02720ZZ, 02723ZZ, 02724ZZ, 02730ZZ, 02733ZZ, 02734ZZ, 02C00ZZ, 02C03ZZ, 02C04ZZ, 02C10ZZ, 02C13ZZ, 02C14ZZ, 02C20ZZ, 02C23ZZ, 02C24ZZ, 02C30ZZ, 02C33ZZ, 02C34ZZ, 3E07017, 3E070PZ, 3E07317, 3E073PZ; NHI procedure code: 68023B, 68024B, 68025B, 68053B, 68054B, 68055B, 3076B, 33077B, 33078B, N26002, N26003 |
| Venous thromboembolism | ICD 9: 451-453, 415.1; ICD 10: I80-I82 |
| Pulmonary disease |  |
| Asthma | ICD 9: 493; ICD 10: J45 |
| Chronic obstructive pulmonary disease | ICD 9: 491, 492, 496; ICD 10: J41, J42, J43, J44 |
| Pneumonia | ICD 9: 480-486; ICD 10: J12-J18 |
| Psychiatric disorders |  |
| Depression | ICD 9: 296.2, 296.3, 300.4, 311; ICD 10: F32-33, F34. |
| Anxiety | ICD 9: 300; ICD 10: F40, F41 |
| Schizophrenia | ICD 9: 295; ICD 10: F20, F2 |
| Neurologic disorders |  |
| Dementia | ICD 9: 290, 331; ICD 10: G30, F00, F01, F03, F05.1, G31.1, G31.82, G31.9 |
| Epilepsy | ICD 9: 345; ICD 10: G40 |
| Bone and joint disorders |  |
| Fracture | ICD 9: 800-829; ICD 10: S12, S22, S32, S42, S52, S62, S72, S82, S92, T02, T08, T10, T12 |
| Osteoporosis | ICD 9: 733.0; ICD 10: M81.0 |
| Osteoarthritis | ICD 9: 715; ICD 10: M15-m19 |
| Anemia | ICD 9: 280-285; ICD 10: D46.1, D46.4, D50-D64 |
| Thyroid disease | ICD 9: 240-246; ICD 10: E00-E07, E35, E89.0 |
| Liver disease | ICD 9: 571; ICD 10: K70-76 |
| Chronic kidney disease | ICD 9: 250.4, 274.1, 283.11, 403.1, 404.2, 404.3, 440.1, 442.1, 447.3, 572.3, 580-588, 642.1, and 646.2; ICD-10-CM codes: N18.x, N08, N19, I12, I13, E10.2, E11.2, E13.2 and E14.2 |
| Hyperkalemia | ICD 9: 276.7; ICD 10: E87.5 |
| Hypokalemia | ICD 9: 276.8; ICD 10: E87.6 |
| Autoimmune diseases | ICD 9: 099.3, 135, 136.1, 255.4, 287.31, 335.2, 340, 358.0, 374.53, 379.0, 390-392, 393-398, 416, 446.0, 446.4-5, 555, 556.9, 576.1, 579.0, 695.2, 695.4, 696.0-1, 701.0  ICD 10: M02.30, D086, M35.2, E27.1-E27.6, E89.6, D69.3, D69.4, G12.2, G12.8, G35, G70.0, H02.73, H15.0, H15.1, I00-I02, I05-I09, I27, M30, M31.3, M31.5-M31.7, K50, K51.9, K83.0, K90.0, L52, L93, L40, L90.0, L94.0, L94.1, L94.3 |
| Cancer | ICD 9: 140-208, 230-234; ICD 10: C00-C97 |
| GERD | ICD 9: 530.11, 530.81; ICD 10: K21 |
| **Co-medications** | **Individual drugs or ATC codes** |
| Biguanides | Buformin and metformin |
| Thiazolidinediones | Pioglitazone and rosiglitazone |
| Alpha-glucosidase inhibitors | Acarbose and miglitol |
| Sodium glucose co-transport-2 inhibitors | Canagliflozin, dapagliflozin, empagliflozin and ertugliflozin |
| Insulin | Insulin aspart, insulin glulisine, insulin human regular, insulin lispro, Insulin human NPH,  Insulin aspart 70/30, insulin lispro 50/50, insulin lispro 75/25, insulinNPH/regular 70/30, Insulin degludec, insulin detemir and insulin glargine |
| Glucagon-like peptide-1 receptor agonist | Exenatide, Lixisenatide, Dulaglutide, Liraglutide and Semaglutide |
| Angiotensin-converting enzyme inhibitor | Benazepril, captopril, cilazapril, enalapril, fosinopril, imidapril, lisinopril, perindopril, quinapril and ramipril |
| Angiotensin receptor blockers | Azilsartan, candesartan, eprosartan, irbesartan, losartan, olmesartan, telmisartan and valsartan |
| Alpha-agonists | Methyldopa |
| Alpha-blockers | Prazosin and doxazosin |
| Beta-blockers | Acebutolol, alprenolol, atenolol, betaxolol, bisoprolol, bupranolol, carvedilol, esmolol, metipranolol metoprolol, labetalol, levobunolol, nadolol, oxprenolol, pindolol, propranolol, sotalol and timolol |
| Sacubitril | Sacubitril |
| Ivabradine | Ivabradine |
| Calcium channel blockers | Amlodipine, barnidipine, benidipine, diltiazem, felodipine, isradipine, lacidipine, lercanidipine, nicardipine, nifedipine, nimodipine, nisoldipine, nitrendipine, and verapamilc |
| Dihydropyridines | Amlodipine, barnidipine, benidipine, felodipine, isradipine, lacidipine, lercanidipine, nicardipine, nifedipine, nimodipine, nisoldipine and nitrendipine |
| Non-dihydropyridines | Diltiazem and verapamil |
| Diuretics | Amiloride, bendroflumethiazide, benzylhydrochlorothiazide, bumetanide, canrenoate, clopamide, eplerenone, ethacrynic acid, furosemide, hydralazine hydrochlorothiazide, hydroflumethiazide, indapamide, metolazone, spironolactone, thiabutazide, triamterene, trichlormethiazide, cyclopenthiazide, and clofenamide |
| Thiazides | Amiloride, bendroflumethiazide, benzylhydrochlorothiazide, clofenamide, clopamide, cyclopenthiazide, hydrochlorothiazide, hydroflumethiazide, indapamide, metolazone, , thiabutazide and trichlormethiazide |
| Loop | Bumetanide, ethacrynic acid and furosemide |
| Potassium-sparing agents | Eplerenone, potassium canrenoate, spironolactone and triamterene |
| Antiplatelets | Abciximab, acetylsalicylic acid, aspirin, cilostazol, clopidogrel, dipyridamole, epoprostenol, eptifibatide, iloprost, prasugrel, selexipag, ticagrelor, ticlopidine, tirofiban and treprostinil |
| Anticoagulants | Heparin, warfarin, urokinase, dalteparin, rivaroxaban, phenindione, streptokinase, nadroparine, enoxaparin, tinzaparin, fondaparinux, dabigatran, apixaban, edoxaban, tenecteplase, protein c and alteplase |
| Statins | Atorvastatin, fluvastatin, lovastatin, pitavastatin, pravastatin, rosuvastatin and simvastatin |
| Others lipid-lowering agents | Acipimox, alirocumab, bezafibrate, cholestyramine, clofibrate, colestipol, etofibrate, evolocumab, ezetimibe, fenofibrate, gemfibrozil, niacin, niceritrol, nicofuranose, nicomol, probucol and simfibrate |
| Nitrates | Nitroglycerin, isosorbide dinitrate, pentaerythritol tetranitrate and isosorbide 5-mononitrtate |
| Antiarrhythmics | Adenosine, amiodarone, disopyramide, dronedarone, flecainide, ivabradine, lidocaine, mexiletine, prajmaline, procainamide, propafenone, quinidine, rauwolfia serpentina, rescinnamine, reserpine, sparteine and ubidecarenone |
| Digoxin | Digoxin |
| Erythropoietin Stimulating Agents | Epoetin alfa, methoxy polyethylene glycol-epoetin beta, epoetin beta, recombinant human erythropoietin and darbepoetin alfa |
| NSAIDs | Aceclofenac, acemetacin, alclofenac, alminoprofen, benzydamine, celecoxib, diclofenac, etodolac, etoricoxib, fenbufen, fenoprofen, flufenamate aluminum, flurbiprofen, glucosamine, ibuprofen, indomethacin, ketoprofen, ketorolac, meclofenamate, mefenamic acid, meloxicam, mepirizole, nabumetone, naproxen, niflumic acid, nimesulide, phenylbutazone, piroxicam, piroxicam, rofecoxib, sulindac, tenoxicam, tiaprofenic acid, tolfenamic acid and tolmetin |
| Steroids | Betamethasone, cortisone, dexamethasone, fludrocortisone, hydrocortisone, methylprednisolone, paramethasone, prednisolone and triamcinolone |
| K channel opener | Nicorandil, diazoxide |
| Inhibitors of mitochondrial PT pore | Cyclosporin A, adenosine, opioids |
| Proton pump inhibitors | Dexlansoprazole, esomeprazole, lansoprazole, omeprazole, pantoprazole and rabeprazole |
| Anticonvulsants | Brivaracetam, carbamazepine, clonazepam, dipropylacetamide, gabapentin, lacosamide, lamotrigine, levetiracetam, oxcarbazepine, perampanel, phenobarbital, phenytoin, pregabalin, primaclone, rufinamide, tiagabine, topiramate, valproate, vigabatrin and zonisamide |
| Antidepressants | Agomelatine, amitriptyline, bupropion, citalopram, clomipramine, dothiepin, doxepin, duloxetine, escitalopram, fluoxetine, fluvoxamine, imipramine, maprotiline, mianserin, milnacipran, mirtazapine, moclobemide, oxitriptan, paroxetine, sertraline, trazodone, venlafaxine, viloxazine and vortioxetine |
| Antipsychotics | Amisulpride, aripiprazole, brexpiprazole, chlorpromazine, chlorprothixene, clopenthixol, clotiapine, clozapine, droperidol, flupentixol, fluphenazine, haloperidol, lithium, loxapine, lurasidone, methotrimeprazine, moperone, olanzapine, paliperidone, penfluridol, perphenazine, pimozide, pipotiazine, prochlorperazine, quetiapine, risperidone, sulpiride, thioridazine, thiothixene, trifluoperazine, ziprasidone and zotepine |
| **Healthcare utilization** | **Operational definition** |
| HbA1c test | NHI procedure codes 09006C |
| Serum creatinine tests | NHI procedure codes 09015C |
| Lipid tests | NHI procedure codes 09001C, 09001CA, 09004C, 09044C, 09043C |
| Electrocardiograms | NHI procedure codes 18001C |
| Echocardiography | NHI procedure codes 18005B, 18005C, 18006B, 18006C, 18044B |

Abbreviations: DM = diabetes mellitus; MI = myocardial infarction CV = cardiovascular; GERD = gastroesophageal reflux disease; NSAIDs = nonsteroidal anti-inflammatory drugs; HbA1c, Glycated hemoglobin; BNP, Brain natriuretic peptide; proBNP, Pro-brain natriuretic peptide; NT-proBNP, N-terminal pro-brain natriuretic peptide.

# e-Table-2. Sensitivity analysis and their purposes

| **Sensitivity Analysis Item** | **Purpose** |
| --- | --- |
| Redefined 3-point major adverse cardiovascular events (3P-MACE) by including both primary and secondary diagnoses | To evaluate whether a broader diagnostic definition of MACE captures additional clinically relevant major adverse cardiovascular events and affects the estimated treatment effects. |
| Excluded patients with prior myocardial infarction (MI) or stroke hospitalization in the previous year before cohort entry | To reduce potential confounding by underlying cardiovascular risk, ensuring that prior events do not influence the observed outcomes. |
| Conducted one-year and two-year intent-to-treat analyses based on the initial treatment among the study cohort | To account for potential informative censoring due to treatment switching or discontinuation. |
| Redefined continuous use using 30-day and 90-day refill grace periods | To assess the impact of different definitions of treatment continuity. |
| Initiated truncation of stabilized propensity score-based inverse probability treatment weighting (IPTW) weights greater than ten (which represented only 0.12% across three groups) | To reduce the influence of extreme weights and enhance the robustness of the estimates derived from the IPTW approach. |
| Restricted patients to those with medication possession ratio (MPR) ≥ 0.8 | To address potential confounding from different medication adherence between groups. |
| Considered non-cardiovascular mortality as a competing event for the 3P-MACE of MI and ischemic stroke | To account for the competing risk of non-cardiovascular death events, particularly when assessing the outcome of MI or ischemic stroke. |
| Repeated analysis among patients who started metformin and then added sulfonylureas or DPP-4 inhibitors | To address time-lag bias by aligning patient pathways through initiating metformin—commonly used as first-line antidiabetic therapy—and ensuring similar treatment escalation patterns, thereby minimizing bias due to differences in treatment timing. |
| Adjusted for hypoglycemic events during follow-up | To examine whether hypoglycemia mediates the relationship between sulfonylurea use and increased cardiovascular risk. |
| Employed gastroesophageal reflux disease (GERD) as a negative control outcome | To assess unmeasured confounding, we used GERD as a negative outcome, as it is unrelated to the exposures of interest, with the expectation of observing a null association if the study design is valid. |
| Examined hypoglycemia as a positive control outcome | To validate the study design by confirming the known higher risk of hypoglycemia with sulfonylureas compared to DPP-4 inhibitors. |

# e-Table 3. The mean duration and reasons for truncation during follow-up within each group by outcomes

| **Outcomes** | **3-point MACE^*^** | | | **Myocardial infarction** | | | **Ischemic stroke** | | |
| --- | --- | --- | --- | --- | --- | --- | --- | --- | --- |
|  | **MitoK_ATP_ channel-low affinity SU (n = 392,319)** | **MitoK_ATP_ channel-high affinity SU (n = 72,817)** | **DPP4**  **inhibitors**  **(n = 309,152)** | **MitoK_ATP_ channel- low affinity SU (n = 392,319)** | **MitoK_ATP_ channel- low affinity SU (n = 392,319)** | **DPP4**  **inhibitors (n = 309,152)** | **MitoK_ATP_ channel-low affinity SU**  **(n = 392,319)** | **MitoK_ATP_ channel-high affinity SU**  **(n = 72,817)** | **DPP4**  **inhibitors (n = 309,152)** |
| **Follow-up time,**  mean±SD (month) | 19.1±23.4 | 16.4±18.9 | 21.9±22.8 | 19.3±23.5 | 16.5±19.0 | 22.1±22.8 | 19.2±23.5 | 16.4±18.9 | 22.0±22.8 |
| **Follow-up**, median (25th, 75th, IQR), month | 8.9 (3.6, 25.6) | 8.3 (5.5, 19.0) | 13.7 (4.8, 30.4) | 9.0 (3.6. 25.8) | 8.4 (5.6, 19.1) | 13.7 (4.9, 30.7) | 9.0 (3.6, 25.7) | 8.4 (5.6, 19.0) | 13.7 (4.9, 30.5) |
| **Censorship, %** |  |  |  |  |  |  |  |  |  |
| Discontinuation | 50.85 | 46.68 | 48.8 | 51.22 | 47.18 | 49.16 | 50.97 | 46.98 | 48.97 |
| Switching/ Add-on | 31.01 | 39.25 | 25.59 | 31.41 | 39.86 | 26.00 | 31.2 | 39.56 | 25.82 |
| Pregnancy | 0.06 | 0.06 | 0.07 | 0.06 | 0.06 | 0.07 | 0.06 | 0.06 | 0.07 |
| Death | 0.99 | 1.06 | 0.96 | 1.25 | 1.24 | 1.2 | 1.26 | 1.22 | 1.2 |
| Disenrollment | 2.82 | 2.61 | 3.25 | 3.34 | 3.17 | 3.88 | 3.32 | 3.12 | 3.85 |
| End of study | 12.2 | 8.01 | 18.98 | 12.29 | 8.04 | 19.2 | 12.24 | 8.01 | 19.05 |
| Outcomes | 2.08 | 2.33 | 2.34 | 0.42 | 0.44 | 0.48 | 0.96 | 1.04 | 1.04 |

Abbreviations: MACE, major adverse cardiovascular events; MitoK_ATP_, mitochondrial ATP-sensitive potassium channel; SU, sulfonylurea; DPP-4 inhibitors, dipeptidyl peptidase-4 inhibitors; SD, standard deviation.

^*^3-point MACE includes myocardial infarction, ischemic stroke, and cardiovascular death.

# e-Table 3. The mean duration and reasons for truncation during follow-up within each group by outcomes (continued)

| **Outcomes** | **Cardiovascular death** | | | | **Heart failure** | | | **Arrhythmia** | | |
| --- | --- | --- | --- | --- | --- | --- | --- | --- | --- | --- |
|  | | **MitoK_ATP_ channel-low affinity SU**  **(n = 392,319)** | **MitoK_ATP_ channel-high affinity SU (n = 72,817)** | **DPP4**  **inhibitors (n = 309,152)** | **MitoK_ATP_ channel-low affinity SU**  **(n = 392,319)** | **MitoK_ATP_ channel-high affinity SU (n = 72,817)** | **DPP4**  **inhibitors (n = 309,152)** | **MitoK_ATP_ channel-low affinity SU**  **(n = 392,319)** | **MitoK_ATP_ channel-high affinity SU**  **(n = 72,817)** | **DPP4**  **inhibitors (n = 309,152)** |
| **Follow-up time,**  mean±SD (month) | | 17.5±22.0 | 15.9±17.5 | 22.1±24.2 | 17.5±22.0 | 15.9±17.5 | 22.0±24.2 | 17.5±22.0 | 15.9±17.5 | 22.0±24.2 |
| **Follow-up**, median (25th, 75th, IQR), month | | 8.3 (3.1, 23.3) | 8.6 (5.7, 19.5) | 13.7 (4.6, 30.5) | 8.2 (3.1, 23.3) | 8.6 (5.7, 19.4) | 13.7 (4.5, 30.3) | 8.2 (3.1, 23.3) | 8.6 (5.7, 19.5) | 13.7 (4.6, 30.4) |
| **Censorship, %** | |  |  |  |  |  |  |  |  |  |
| Discontinuation | | 51.34 | 47.12 | 49.34 | 51.16 | 47.17 | 49.02 | 51.27 | 47.34 | 49.23 |
| Switching/ Add-on | | 31.6 | 39.83 | 25.88 | 31.42 | 39.8 | 25.96 | 31.53 | 39.96 | 26.07 |
| Pregnancy | | 0.06 | 0.06 | 0.07 | 0.06 | 0.06 | 0.07 | 0.06 | 0.06 | 0.07 |
| Death | | 0.73 | 0.82 | 0.72 | 1.26 | 1.21 | 1.18 | 1.27 | 1.24 | 1.22 |
| Disenrollment | | 2.23 | 2.17 | 2.7 | 3.31 | 3.15 | 3.81 | 3.37 | 3.19 | 3.94 |
| End of study | | 12.33 | 8.05 | 19.27 | 12.31 | 8.02 | 19.17 | 12.31 | 8.04 | 19.2 |
| Outcomes | | 1.7 | 1.94 | 2.02 | 0.48 | 0.59 | 0.79 | 0.18 | 0.17 | 0.26 |

Abbreviations: MitoK_ATP_, mitochondrial ATP-sensitive potassium channel; SU, sulfonylurea; DPP-4 inhibitors, dipeptidyl peptidase-4 inhibitors; SD, standard deviation.

# e-Table 3. The mean duration and reasons for truncation during follow-up within each group by outcomes (continued)

| **Outcomes** | **Severe hypoglycemia** | | | **All-cause mortality** | | |
| --- | --- | --- | --- | --- | --- | --- |
|  | **MitoK_ATP_ channel-low affinity sulfonylureas**  **(n = 392,319)** | **MitoK_ATP_ channel-high affinity sulfonylureas**  **(n = 72,817)** | **DPP4i**  **(n = 309,152)** | **MitoK_ATP_ channel-low affinity sulfonylureas**  **(n = 392,319)** | **MitoK_ATP_ channel-high affinity sulfonylureas**  **(n = 72,817)** | **DPP4i**  **(n = 309,152)** |
| **Follow-up time,**  mean±SD (month) | 17.5±22.0 | 15.9±17.5 | 22.1±24.2 | 17.5±22.0 | 15.9±17.5 | 22.0±24.2 |
| **Follow-up**, median (25th, 75th, IQR), month | 8.2 (3.1, 23.3) | 8.6 (5.7, 19.5) | 13.7 (4.6, 30.5) | 8.3 (3.1, 23.3) | 8.6 (5.7, 19.5) | 13.6 (4.5, 30.5) |
| **Censorship, %** |  |  |  |  |  |  |
| Discontinuation | 51.22 | 47.19 | 49.29 | 51.34 | 46.65 | 49.33 |
| Switching/ Add-on | 31.49 | 39.91 | 26.08 | 31.6 | 39.48 | 25.67 |
| Pregnancy | 0.06 | 0.06 | 0.07 | 0.06 | 0.06 | 0.07 |
| Death | 1.27 | 1.24 | 1.23 | N/A | N/A | N/A |
| Disenrollment | 3.37 | 3.18 | 3.95 | 0.77 | 0.56 | 0.87 |
| End of study | 12.33 | 8.05 | 19.26 | 12.33 | 8.05 | 19.27 |
| Outcomes | 0.27 | 0.36 | 0.12 | 3.9 | 5.2 | 4.78 |

Abbreviations: MitoK_ATP_, mitochondrial ATP-sensitive potassium channel; SU, sulfonylurea; DPP-4 inhibitors, dipeptidyl peptidase-4 inhibitors; SD, standard deviation.

# e-Table 4. Demographic and clinical characteristics of users of MitoK_ATP_ channel-low affinity sulfonylureas, MitoK_ATP_ channel-high affinity sulfonylureas, and DPP-4 inhibitors before propensity score-based IPTW

| **Characteristics*** | **MitoK_ATP_ channel-low affinity sulfonylureas**  **(n =** **466,158)** | **MitoK_ATP_ channel-high affinity sulfonylureas**  **(n = 83,031)** | **DPP-4 inhibitors (n = 473,539)** | **MAX aSMD^†^** |
| --- | --- | --- | --- | --- |
| Age, mean (SD) | 58.3 (13.2) | 59.6 (13.6) | 62.2 (14.2) | 0.296 |
| Sex, male No. (%) | 268,553 (57.6) | 48,163 (58) | 25,1895 (53.2) | 0.096 |
| Period from the first DM diagnosis to the initial use of medication (month), mean (SD) | 10.7 (19.5) | 9.0 (17.5) | 22.1 (27.6) | 0.606 |
| Entry year |  |  |  |  |
| 2013 | 72,631 (15.6) | 18,785 (22.6) | 37,889 (8.0) | 0.432 |
| 2014 | 75,828 (16.3) | 17,041 (20.5) | 43,850 (9.3) | 0.280 |
| 2015 | 63,542 (13.6) | 11,628 (14.0) | 45,516 (9.6) | 0.117 |
| 2016 | 53,360 (11.5) | 8,840 (10.7) | 45,246 (9.6) | 0.066 |
| 2017 | 48,799 (10.5) | 7,259 (8.7) | 49,652 (10.5) | 0.048 |
| 2018 | 45,239 (9.7) | 6,367 (7.7) | 56,715 (12) | 0.137 |
| 2019 | 40,095 (8.6) | 5,260 (6.3) | 62,063 (13.1) | 0.223 |
| 2020 | 36,467 (7.8) | 4,484 (5.4) | 67,333 (14.2) | 0.300 |
| 2021 | 30,197 (6.5) | 3,367 (4.1) | 65,275 (13.8) | 0.346 |
| **Monthly income-based insurance premium (NTD), No. (%)** | | |  |  |
| First tertial | 188,166 (40.4) | 37,124 (44.7) | 191,557 (40.5) | 0.090 |
| Second tertial | 112,130 (24.1) | 19,074 (23.0) | 102,745 (21.7) | 0.058 |
| Third tertial | 165,862 (35.6) | 26,833 (32.3) | 179,237 (37.9) | 0.123 |
| **Diabetes severity indicators** |  |  |  |  |
| aDCSI, Mean (SD) | 0.6 (1.2) | 0.8 (1.3) | 1.1 (1.6) | 0.362 |
| aDCSI score, No (%) |  |  |  |  |
| 0 | 317,643 (68.1) | 53,624 (64.6) | 247,013 (52.2) | 0.331 |
| 1 | 71,223 (15.3) | 12,257 (14.8) | 86,342 (18.2) | 0.094 |
| 2 | 41,626 (8.9) | 8,531 (10.3) | 60,663 (12.8) | 0.125 |
| 3+ | 35,666 (7.7) | 8619 (10.4) | 79,521 (16.8) | 0.282 |
| Hypoglycemia, No. (%) | 2,455 (0.5) | 678 (0.8) | 3921 (0.8) | 0.040 |
| Diabetes treatment, No. (%) |  |  |  |  |
| Antidiabetics drugs at cohort entry date | |  |  |  |
| 1 | 111,777 (24.0) | 19,339 (23.3) | 123,692 (26.1) | 0.058 |
| 2 | 291,206 (62.5) | 51,976 (62.6) | 273,844 (57.8) | 0.022 |
| 3+ | 63,175 (13.6) | 11,716 (14.1) | 76,003 (16.1) | 0.090 |
| Diabetes drug on cohort entry date | |  |  |  |
| Biguanides | 335,994 (72.1) | 59,399 (71.5) | 301,647 (63.7) | 0.111 |
| Thiazolidinediones | 18,223 (3.9) | 1,997 (2.4) | 12,001 (2.5) | 0.058 |
| Alpha-glucosidase inhibitors | 17,827 (3.8) | 3,303 (4) | 19,714 (4.2) | 0.034 |
| Meglitinides | 2,901 (0.6) | 887 (1.1) | 31,664 (6.7) | 0.337 |
| SGLT2i | 11,319 (2.4) | 845 (1.0) | 3,638 (0.8) | 0.094 |
| GLP-1 RA^‡^ | 1,064 (0.2) | 115 (0.1) | 108 (0.02) | 0.062 |
| Insulin | 38,084 (8.2) | 10,174 (12.3) | 68,810 (14.5) | 0.203 |
| Diabetes drug in baseline |  |  |  |  |
| Biguanides | 259,904 (55.8) | 41,978 (50.6) | 300,066 (63.4) | 0.400 |
| Thiazolidinediones | 16,477 (3.5) | 2,183 (2.6) | 19,482 (4.1) | 0.116 |
| Alpha-glucosidase inhibitors | 21,853 (4.7) | 3,853 (4.6) | 29,728 (6.3) | 0.103 |
| Meglitinides | 20,476 (4.4) | 4,100 (4.9) | 38,096 (8.0) | 0.129 |
| SGLT2i | 10,270 (2.2) | 864 (1.0) | 12,466 (2.6) | 0.157 |
| GLP-1 RA | 780 (0.2) | 75 (0.1) | 647 (0.1) | 0.018 |
| Insulin | 41,915 (9.0) | 9,346 (11.3) | 78,518 (16.6) | 0.250 |
| **Health care utilization, No. (%)** |  |  |  |  |
| Hospital level |  |  |  |  |
| Academic medical centers | 39,267 (8.4) | 7,683 (9.3) | 81,374 (17.2) | 0.265 |
| Metropolitan hospitals | 68,123 (14.6) | 12,903 (15.5) | 110,045 (23.2) | 0.222 |
| Local community hospitals | 39,340 (8.4) | 9,701 (11.7) | 63,620 (13.4) | 0.161 |
| Physician clinics | 319,428 (68.5) | 52,744 (63.5) | 218,500 (46.1) | 0.465 |
| Hospitalizations |  |  |  |  |
| 0 | 394,753 (84.7) | 66,930 (80.6) | 365,902 (77.3) | 0.182 |
| 1 | 51,591 (11.1) | 10,790 (13.0) | 72,086 (15.2) | 0.119 |
| ≥2 | 19,814 (4.3) | 5,311 (6.4) | 35,551 (7.5) | 0.132 |
| Emergency department visits |  |  |  |  |
| 0 | 350,498 (75.2) | 59,987 (72.3) | 325,283 (68.7) | 0.136 |
| 1 | 78,098 (16.8) | 14,531 (17.5) | 90,894 (19.2) | 0.058 |
| ≥2 | 37,562 (8.1) | 8,513 (10.3) | 57,362 (12.1) | 0.129 |
| Internal medicine visits |  |  |  |  |
| 0 | 233,415 (50.1) | 39,654 (47.8) | 249,842 (52.8) | 0.093 |
| 1 | 58,850 (12.6) | 10,674 (12.9) | 60,296 (12.7) | 0.011 |
| ≥2 | 173,893 (37.3) | 32,703 (39.4) | 163,401 (34.5) | 0.089 |
| Endocrinologist visits |  |  |  |  |
| 0 | 394,327 (84.6) | 72,466 (87.3) | 334,015 (70.5) | 0.445 |
| 1 | 23,482 (5.0) | 3,260 (3.9) | 27,013 (5.7) | 0.074 |
| ≥2 | 48,349 (10.4) | 7,305 (8.8) | 112,511 (23.8) | 0.447 |
| Cardiologist visits |  |  |  |  |
| 0 | 393,606 (84.4) | 69,275 (83.4) | 334,569 (70.7) | 0.334 |
| 1 | 14,859 (3.2) | 2,832 (3.4) | 21,028 (4.4) | 0.064 |
| ≥2 | 57,693 (12.4) | 10,924 (13.2) | 117,942 (24.9) | 0.326 |
| Nephrologist visits |  |  |  |  |
| 0 | 445,843 (95.6) | 78,289 (94.3) | 422,297 (89.2) | 0.247 |
| 1 | 7,316 (1.6) | 1,569 (1.9) | 12,404 (2.6) | 0.071 |
| ≥2 | 12,999 (2.8) | 3,173 (3.8) | 38,838 (8.2) | 0.242 |
| HbA1C tests ordered |  |  |  |  |
| 0 | 115,590 (24.8) | 25,723 (31.0) | 51,122 (10.8) | 0.534 |
| 1 | 147,493 (31.6) | 24,424 (29.4) | 106,304 (22.5) | 0.203 |
| ≥2 | 203,075 (43.6) | 32,884 (39.6) | 316,113 (66.8) | 0.594 |
| Serum creatinine tests ordered |  |  |  |  |
| 0 | 144,513 (31.0) | 26,814 (32.3) | 57,541 (12.2) | 0.511 |
| 1 | 133,749 (28.7) | 22,041 (26.6) | 111,025 (23.5) | 0.095 |
| ≥2 | 187,896 (40.3) | 34,176 (41.2) | 304,973 (64.4) | 0.493 |
| Lipid tests ordered |  |  |  |  |
| 0 | 150,558 (32.3) | 31,287 (37.7) | 74,193 (15.7) | 0.072 |
| 1 | 131,778 (28.3) | 21,933 (26.4) | 112,101 (23.7) | 0.065 |
| ≥2 | 183,822 (39.4) | 29,811 (35.9) | 287,245 (60.7) | 0.031 |
| Electrocardiograms tests ordered | |  |  |  |
| 0 | 327,376 (70.2) | 56,028 (67.5) | 274,907 (58.1) | 0.216 |
| 1 | 95,193 (20.4) | 17,227 (20.8) | 122,982 (26.0) | 0.128 |
| ≥2 | 43,589 (9.4) | 9,776 (11.8) | 75,650 (16.0) | 0.157 |
| **Comorbidities, No. (%)** |  |  |  |  |
| Cardiovascular disease |  |  |  |  |
| Myocardial infraction |  |  |  |  |
| None | 463,344 (99.4) | 82,442 (99.3) | 467,691 (98.8) | 0.056 |
| History | 1,495 (0.3) | 305 (0.4) | 3,155 (0.7) | 0.108 |
| Hospitalization | 1,319 (0.3) | 284 (0.3) | 2,693 (0.6) | 0.039 |
| Ischemic stroke |  |  |  |  |
| None | 455,212 (97.7) | 80,257 (96.7) | 456,974 (96.5) | 0.073 |
| History | 8,955 (1.9) | 2,286 (2.8) | 14,010 (3.0) | 0.070 |
| Hospitalization | 1,991 (0.4) | 488 (0.6) | 2,555 (0.5) | 0.026 |
| Heart failure |  |  |  |  |
| None | 451,668 (96.9) | 80,041 (96.4) | 444,666 (93.9) | 0.138 |
| History | 11,334 (2.4) | 2,154 (2.6) | 20,824 (4.4) | 0.116 |
| Hospitalization | 3,156 (0.7) | 836 (1.0) | 8,049 (1.7) | 0.081 |
| Cardiac arrhythmia |  |  |  |  |
| None | 446,944 (95.9) | 79,089 (95.3) | 442,076 (93.4) | 0.067 |
| History | 16,659 (3.6) | 3,231 (3.9) | 25,746 (5.4) | 0.092 |
| Hospitalization | 2,555 (0.6) | 711 (0.9) | 5,717 (1.2) | 0.067 |
| Hemorrhagic stroke | 4,089 (0.9) | 981 (1.2) | 6,650 (1.4) | 0.047 |
| Other stroke | 16,128 (3.5) | 3,872 (4.7) | 21,141 (4.5) | 0.054 |
| Ischemic heart disease | 47,864 (10.3) | 8,954 (10.8) | 73,876 (15.6) | 0.164 |
| Coronary revascularization | 1,805 (0.4) | 362 (0.4) | 3,557 (0.8) | 0.048 |
| Hypertension | 240,492 (51.6) | 42,153 (50.8) | 278,241 (58.8) | 0.178 |
| Dyslipidemia | 212,020 (45.5) | 31,147 (37.5) | 241,612 (51.0) | 0.320 |
| Peripheral arterial disease | 7,683 (1.7) | 1,486 (1.8) | 9,957 (2.1) | 0.039 |
| Venous thromboembolism | 1,603 (0.3) | 371 (0.5) | 3,008 (0.6) | 0.042 |
| Pulmonary diseases |  |  |  |  |
| Asthma | 21,344 (4.6) | 3,971 (4.8) | 24,771 (5.2) | 0.025 |
| COPD | 22,409 (4.8) | 5,085 (6.1) | 30,198 (6.4) | 0.064 |
| Pneumonia | 18,538 (4) | 4,461 (5.4) | 27,509 (5.8) | 0.076 |
| Psychiatric disorder |  |  |  |  |
| Depression | 17,321 (3.7) | 3,324 (4.0) | 21,371 (4.5) | 0.037 |
| Anxiety | 39,829 (8.5) | 7,123 (8.6) | 41,970 (8.9) | 0.009 |
| Schizophrenia | 4,831 (1.0) | 1,129 (1.4) | 4,400 (0.9) | 0.044 |
| Neurologic disorders |  |  |  |  |
| Dementia | 8,872 (1.9) | 2,408 (2.9) | 19,034 (4.0) | 0.122 |
| Epilepsy | 2,901 (0.6) | 689 (0.8) | 4,146 (0.9) | 0.029 |
| Bone and joint disorders |  |  |  |  |
| Fracture | 21,935 (4.7) | 4,642 (5.6) | 27,959 (5.9) | 0.054 |
| Osteoporosis | 8,057 (1.7) | 1,825 (2.2) | 12,696 (2.7) | 0.064 |
| Osteoarthritis | 64,956 (13.9) | 12,253 (14.8) | 80,324 (17) | 0.081 |
| Anemia | 14,551 (3.1) | 2,981 (3.6) | 20,928 (4.4) | 0.068 |
| Thyroid disease | 15,443 (3.3) | 2,393 (2.9) | 24,990 (5.3) | 0.124 |
| Liver disease | 57,837 (12.4) | 9,939 (12.0) | 56,079 (11.8) | 0.013 |
| Chronic kidney disease | 51,702 (11.1) | 11,570 (13.9) | 102,049 (21.6) | 0.304 |
| Hyperkalemia | 773 (0.2) | 281 (0.3) | 2,693 (0.6) | 0.068 |
| Hypokalemia | 3,050 (0.7) | 795 (1.0) | 4,536 (1.0) | 0.031 |
| Autoimmune diseases | 9,340 (2.0) | 1,971 (2.4) | 11,663 (2.5) | 0.024 |
| Cancer | 13,234 (2.8) | 3,326 (4.0) | 19,942 (4.2) | 0.080 |
| GERD | 27,502 (5.9) | 4,592 (5.5) | 40,373 (8.5) | 0.104 |
| **Co-medication, No. (%)** |  |  |  |  |
| Cardiovascular comedication |  |  |  |  |
| ACEIs | 27,575 (5.9) | 5,803 (7.0) | 27,326 (5.8) | 0.037 |
| ARBs | 148,774 (31.9) | 23,199 (27.9) | 205,344 (43.4) | 0.343 |
| Alpha-blockers | 3,139 (0.7) | 652 (0.8) | 3,523 (0.7) | 0.008 |
| Beta-blockers | 105,857 (22.7) | 19,414 (23.4) | 143,369 (30.3) | 0.172 |
| Calcium channel blockers |  |  |  |  |
| Dihydropyridines | 158,007 (33.9) | 28,608 (34.5) | 193,841 (40.9) | 0.155 |
| Non-dihydropyridines | 12,119 (2.6) | 2,565 (3.1) | 18,852 (4.0) | 0.076 |
| Diuretics |  |  |  |  |
| Thiazides | 65,568 (14.1) | 12,265 (14.8) | 73,450 (15.5) | 0.046 |
| Loop | 25,641 (5.5) | 6,257 (7.5) | 48,074 (10.2) | 0.172 |
| Potassium-sparing agents | 9,738 (2.1) | 2,218 (2.7) | 17,715 (3.7) | 0.094 |
| Antiplatelets | 86,017 (18.5) | 16,636 (20) | 131,844 (27.8) | 0.237 |
| Anticoagulants | 10,018 (2.2) | 2,396 (2.9) | 23,436 (5.0) | 0.152 |
| Statins | 144,911 (31.1) | 20,542 (24.7) | 222,018 (46.9) | 0.512 |
| Others lipid-lowering agents | 36,551 (7.8) | 5,853 (7.1) | 39,414 (8.3) | 0.074 |
| Nitrates | 21,392 (4.6) | 4,698 (5.7) | 40,049 (8.5) | 0.166 |
| Antiarrhythmics | 9,374 (2.0) | 2,241 (2.7) | 17,811 (3.8) | 0.101 |
| Digoxin | 4,688 (1.0) | 1,012 (1.2) | 6,620 (1.4) | 0.035 |
| Erythropoietin Stimulating Agents | 1,871 (0.4) | 1,214 (1.5) | 12,187 (2.6) | 0.185 |
| Anti-inflammatory agents |  |  |  |  |
| NSAIDs | 264,543 (56.8) | 46,909 (56.5) | 250,979 (5.03) | 0.081 |
| Steroids | 88,283 (18.9) | 17,113 (20.6) | 99,639 (21.0) | 0.041 |
| K channel opener | 6,152 (1.3) | 1,105 (1.3) | 11,519 (2.4) | 0.086 |
| Inhibitors of mitochondrial PT pore | 111,316 (23.9) | 22,397 (27) | 128,196 (27.1) | 0.062 |
| Proton pump inhibitors | 28,495 (6.1) | 5,929 (7.1) | 45,367 (9.6) | 0.121 |
| Anticonvulsants | 28,060 (6.0) | 5,734 (6.9) | 42,959 (9.1) | 0.115 |
| Antidepressants | 32,443 (7.0) | 6,239 (7.5) | 42,874 (9.1) | 0.076 |
| Antipsychotics | 36,708 (7.9) | 7,879 (9.5) | 46,739 (9.9) | 0.063 |

Abbreviations: DPP4i, dipeptidyl peptidase-4 inhibitor; MitoK_ATP_, mitochondrial ATP-sensitive potassium channel; IPTW, inverse probability of treatment weighting; aSMD, absolute standardized mean difference; No, number; SD, standard deviation; DM, diabetes mellitus; NTD, New Taiwan dollar; aDSCI, adapted Diabetes Complications Severity Index; SGLT2i, sodium-glucose co-transporter 2 inhibitor; HbA1c, glycated hemoglobin; COPD, chronic obstructive pulmonary disease; GERD, gastroesophageal reflux disease; ACEIs, angiotensin converting enzyme inhibitors; ARBs, angiotensin receptor blockers; NSAIDs, nonsteroidal anti-inflammatory drugs; PT, permeability transition.

^*^ All co-medications, diabetes severity indicators, health care utilization, and monthly income were measured in the year preceding the cohort entry date; all comorbidities and diabetes drugs in baseline were measured 180 days preceding the cohort entry date; age, sex, entry year, and hospital level were measured at the cohort entry date.

^†^ Standardized mean difference >0.1 represents meaningful differences between two groups.

^‡^Use of GLP-1RA was not considered in the propensity score estimation model because GLP-1 RA was not allowed to be concomitantly used with DPP-4i based on the reimbursement policy of Taiwan’s national health insurance program.

# e-Table 5. Demographic and clinical characteristics of users of MitoK_ATP_ channel-low affinity sulfonylureas, MitoK_ATP_ channel-high affinity sulfonylureas, and DPP-4 inhibitors after propensity score-based IPTW (Full version)

| **Charatieristics*** | **MitoK_ATP_ channel-low affinity sulfonylureas**  **(n = 484,579)** | **MitoK_ATP_ channel-high affinity sulfonylureas**  **(n =85,729)** | **DPP-4 inhibitors (n = 465,396)** | **MAX aSMD**^†^ |
| --- | --- | --- | --- | --- |
| Age, mean (SD) | 60.8 (14.5) | 60.7 (14.3) | 60.3 (13.8) | 0.071 |
| Sex, male No. (%) | 268,689 (55.5) | 47,451.3 (55.4) | 259,771 (55.8) | 0.033 |
| Period from the first DM diagnosis to the initial use of medication (month), mean (SD) | 16.6 (25.2) | 16.7 (25.5) | 16.2 (24.2) | 0.046 |
| Entry year |  |  |  |  |
| 2013 | 60,190.7 (12.4) | 10,491.5 (12.2) | 55,576.1 (11.9) | 0.032 |
| 2014 | 63,570.8 (13.1) | 11,099.6 (13.0) | 60,568.5 (13.0) | 0.031 |
| 2015 | 56,076.0 (11.6) | 12,120.9 (11.4) | 66,311.2 (11.5) | 0.030 |
| 2016 | 50,640.7 (10.5) | 8,910.1 (10.4) | 49,074.8 (10.5) | 0.029 |
| 2017 | 50,254.0 (10.4) | 8,671.5 (10.1) | 48,383.6 (10.4) | 0.028 |
| 2018 | 50,618.8 (10.5) | 9,188.7 (10.7) | 49,988.8 (10.7) | 0.028 |
| 2019 | 51,501.4 (10.6) | 9,237.0 (10.8) | 50,067.8 (10.8) | 0.028 |
| 2020 | 53,048.8 (11.0) | 9,623.2 (11.2) | 51,357.7 (11.0) | 0.006 |
| 2021 | 48,677.3 (10.1) | 8,725.5 (10.2) | 46,879.2 (10.1) | 0.004 |
| **Monthly income-based insurance premium (NTD), No. (%)** | | |  |  |
| First tertial | 200,247.0 (41.3) | 35,239.7 (41.1) | 189,092.0 (40.6) | 0.027 |
| Second tertial | 108,785.0 (22.5) | 19,312.8 (22.5) | 105,757.0 (22.7) | 0.027 |
| Third tertial | 175,547.0 (36.2) | 31,177.1 (36.4) | 170,547.0 (36.7) | 0.027 |
| **Diabetes severity indicators** |  |  |  |  |
| aDCSI, Mean (SD) | 1.0 (1.6) | 1.0 (1.5) | 0.9 (1.4) | 0.050 |
| aDCSI score, No (%) |  |  |  |  |
| 0 | 283,135.0 (58.4) | 49,827.4 (58.1) | 278,313 (59.8) | 0.019 |
| 1 | 79,471.5 (16.4) | 14,303.9 (16.7) | 77,219.9 (16.6) | 0.026 |
| 2 | 53,865.7 (11.1) | 9,731.9 (11.4) | 51,500.6 (11.1) | 0.028 |
| 3+ | 68,106.1 (14.1) | 11,866.5 (13.8) | 58,361.7 (12.5) | 0.018 |
| Hypoglycemia, No. (%) | 3,931.0 (0.8) | 628.8 (0.7) | 3,273.5 (0.7) | 0.025 |
| Diabetes treatment, No. (%) |  |  |  |  |
| Antidiabetics drugs at cohort entry date | |  |  |  |
| 1 | 117,102.0 (24.2) | 21,554.3 (25.1) | 113,382.0 (24.4) | 0.025 |
| 2 | 283,446.0 (58.5) | 50,343.6 (58.7) | 279,615.0 (60.1) | 0.025 |
| 3+ | 84,030.6 (17.3) | 13,831.7 (16.1) | 72,398.4 (15.6) | 0.024 |
| Diabetes drug on cohort entry date | |  |  |  |
| Biguanides | 327,570.0 (67.6) | 57,572.0 (67.2) | 319,375.0 (68.6) | 0.032 |
| Thiazolidinediones | 15,025.4 (3.1) | 2,699.4 (3.2) | 15,262.2 (3.3) | 0.048 |
| Alpha-glucosidase inhibitors | 20,989.3 (4.3) | 3,496.5 (4.1) | 19,190.1 (4.1) | 0.022 |
| Meglitinides | 27,885.5 (5.8) | 3,586.4 (4.2) | 16,498.1 (3.5) | 0.022 |
| SGLT2i | 7,274.1 (1.5) | 1,345.9 (1.6) | 8,593.5 (1.9) | 0.021 |
| GLP-1 RA^‡^ | 1,667.3 (0.3) | 278.4 (0.3) | 125.4 (0.03) | 0.097 |
| Insulin | 70,333.4 (14.5) | 11,406.5 (13.3) | 56,113.9 (12.1) | 0.027 |
| Diabetes drug in baseline |  |  |  |  |
| Biguanides | 282,211.0 (58.2) | 49,671.8 (57.9) | 274,640.0 (59.0) | 0.074 |
| Thiazolidinediones | 18,373.8 (3.8) | 3,164.1 (3.7) | 17,551.9 (3.8) | 0.072 |
| Alpha-glucosidase inhibitors | 27,494.5 (5.7) | 4,638.8 (5.4) | 25,857.5 (5.6) | 0.021 |
| Meglitinides | 31,282.0 (6.5) | 5,721.0 (6.7) | 29,458.5 (6.3) | 0.021 |
| SGLT2i | 12,372.8 (2.6) | 2,053.8 (2.4) | 11,394.3 (2.5) | 0.020 |
| GLP-1 RA | 772.0 (0.2) | 138.8 (0.2) | 897.6 (0.2) | 0.020 |
| Insulin | 70,011.5 (14.5) | 12,035.9 (14.0) | 60,959.0 (13.1) | 0.020 |
| **Health care utilization, No. (%)** | |  |  |  |
| Hospital level |  |  |  |  |
| Academic medical centers | 65,120.3 (13.4) | 11,754.5 (13.7) | 60,031.5 (12.9) | 0.019 |
| Metropolitan hospitals | 95,106.6 (19.6) | 16,935.3 (19.8) | 89,128.9 (19.2) | 0.039 |
| Local community hospitals | 55,570.1 (11.5) | 9,390.1 (11.0) | 52,068.9 (11.2) | 0.019 |
| Physician clinics | 26,8782.0 (55.5) | 47,649.7 (55.6) | 264,167.0 (56.8) | 0.019 |
| Hospitalizations |  |  |  |  |
| 0 | 383,484.0 (79.1) | 67,936.1 (79.2) | 373,541 (80.3) | 0.018 |
| 1 | 66,896.9 (13.8) | 11,883.5 (13.9) | 62,879.6 (13.5) | 0.045 |
| ≥2 | 34,197.2 (7.1) | 5,910.1 (6.9) | 28,975.4 (6.2) | 0.028 |
| Emergency department visits |  |  |  |  |
| 0 | 340,545.0 (70.3) | 60,472.9 (70.5) | 331,452.0 (71.2) | 0.017 |
| 1 | 88,821.3 (18.3) | 15,593 (18.2) | 85,112.7 (18.3) | 0.033 |
| ≥2 | 55,211.8 (11.4) | 9,663.8 (11.3) | 48,831.6 (10.5) | 0.021 |
| Internal medicine visits |  |  |  |  |
| 0 | 248,149.0 (51.2) | 44,160.9 (51.5) | 239,608.0 (51.5) | 0.017 |
| 1 | 61,710.0 (12.7) | 10,983.3 (12.8) | 59,074.0 (12.7) | 0.029 |
| ≥2 | 174,719.0 (36.1) | 30,585.4 (35.7) | 166,714.0 (35.8) | 0.016 |
| Endocrinologist visits |  |  |  |  |
| 0 | 375,406.0 (77.5) | 66,122.1 (77.1) | 362,274.0 (77.8) | 0.016 |
| 1 | 25,765.8 (5.3) | 4,611.4 (5.4) | 25,293.2 (5.4) | 0.016 |
| ≥2 | 83,406.2 (17.2) | 14,996.1 (17.5) | 77,828.3 (16.7) | 0.016 |
| Cardiologist visits |  |  |  |  |
| 0 | 366,838.0 (75.7) | 65,531.1 (76.4) | 359,091.0 (77.2) | 0.015 |
| 1 | 19,409.9 (4.0) | 3,400.6 (4.0) | 18,450.6 (4.0) | 0.015 |
| ≥2 | 98,331.1 (20.3) | 16,797.9 (19.6) | 87,853.9 (18.9) | 0.034 |
| Nephrologist visits |  |  |  |  |
| 0 | 441,486.0 (91.1) | 78,101.7 (91.1) | 429,598.0 (92.3) | 0.014 |
| 1 | 10,672.0 (2.2) | 1,935.7 (2.3) | 10,009.5 (2.2) | 0.036 |
| ≥2 | 32,420.6 (6.7) | 5,692.3 (6.6) | 25,788.1 (5.5) | 0.044 |
| HbA1C tests ordered |  |  |  |  |
| 0 | 89,619.8 (18.5) | 15,569.9 (18.2) | 86,150.6 (18.5) | 0.013 |
| 1 | 129,628.0 (26.8) | 22,807.9 (26.6) | 125,791 (27.0) | 0.048 |
| ≥2 | 265,331.0 (54.8) | 473,51.9 (55.2) | 253,454 (54.5) | 0.012 |
| Serum creatinine tests ordered | |  |  |  |
| 0 | 103,845.0 (21.4) | 18,317.2 (21.4) | 99,665.1 (21.4) | 0.012 |
| 1 | 122,682.0 (25.3) | 21,611.1 (25.2) | 121,401.0 (26.1) | 0.012 |
| ≥2 | 258,052.0 (53.3) | 45,801.3 (53.4) | 244,330.0 (52.5) | 0.011 |
| Lipid tests ordered |  |  |  |  |
| 0 | 118,617.0 (24.5) | 20,752.0 (24.2) | 114,743.0 (24.7) | 0.018 |
| 1 | 124,647.0 (25.7) | 21,922.9 (25.6) | 120,781.0 (26.0) | 0.015 |
| ≥2 | 241,315.0 (49.8) | 43,054.7 (50.2) | 229,872.0 (49.4) | 0.010 |
| Electrocardiograms tests ordered | |  |  |  |
| 0 | 300,928.0 (62.1) | 53,527.3 (62.4) | 294,916.0 (63.4) | 0.010 |
| 1 | 114,476.0 (23.6) | 20,048.9 (23.4) | 109,487.0 (23.5) | 0.010 |
| ≥2 | 69,174.5 (14.3) | 12,153.4 (14.2) | 60,993.1 (13.1) | 0.026 |
| **Comorbidities, No. (%)** |  |  |  |  |
| Cardiovascular disease |  |  |  |  |
| Myocardial infraction |  |  |  |  |
| None | 479,013.0 (98.9) | 84,928.9 (99.1) | 460,990.0 (99.1) | 0.014 |
| History | 3,224.1 (0.7) | 431.5 (0.5) | 2,364.4 (0.5) | 0.010 |
| Hospitalization | 2,341.3 (0.5) | 369.3 (0.4) | 2,040.9 (0.4) | 0.034 |
| Ischemic stroke |  |  |  |  |
| None | 468,531.0 (96.7) | 82,942.8 (96.8) | 450,895.0 (96.9) | 0.021 |
| History | 13,332.5 (2.8) | 2,335 (2.7) | 12,026.9 (2.6) | 0.009 |
| Hospitalization | 2,714.5 (0.6) | 4,51.9 (0.5) | 2,473.7 (0.5) | 0.011 |
| Heart failure |  |  |  |  |
| None | 458,746.0 (94.7) | 81,359.1 (94.9) | 443,532.0 (95.3) | 0.010 |
| History | 18,005.7 (3.7) | 3,103.5 (3.6) | 16,104.7 (3.5) | 0.009 |
| Hospitalization | 7,826.5 (1.6) | 1,267.1 (1.5) | 5,759.4 (1.2) | 0.029 |
| Cardiac arrhythmia |  |  |  |  |
| None | 455,983.0 (94.1) | 80,723.2 (94.2) | 439,859.0 (94.5) | 0.032 |
| History | 23,309.1 (4.8) | 4,133.2 (4.8) | 21,241.1 (4.6) | 0.020 |
| Hospitalization | 5,286 (1.1) | 873.3 (1.0) | 4,296.2 (0.9) | 0.018 |
| Hemorrhagic stroke | 6,752.2 (1.4) | 1,054.5 (1.2) | 5,576.8 (1.2) | 0.012 |
| Other stroke | 21,580.2 (4.5) | 3,782.7 (4.4) | 19,599.6 (4.2) | 0.017 |
| Ischemic heart disease | 65,654.6 (13.6) | 11,698.7 (13.7) | 60,902.4 (13.1) | 0.017 |
| Coronary revascularization | 3,307.6 (0.7) | 535.4 (0.6) | 2,740.6 (0.6) | 0.012 |
| Hypertension | 268,680.0 (55.5) | 47,762.1 (55.7) | 255,212.0 (54.8) | 0.014 |
| Dyslipidemia | 225,552.0 (46.6) | 40,142.9 (46.8) | 219,048.0 (47.1) | 0.012 |
| Peripheral arterial disease | 9,729.4 (2.0) | 1,704.7 (2.0) | 8,750.6 (1.9) | 0.012 |
| Venous thromboembolism | 2,590.4 (0.5) | 470.6 (0.6) | 2,364.6 (0.5) | 0.010 |
| Pulmonary diseases |  |  |  |  |
| Asthma | 24,572.6 (5.1) | 4,263.3 (5.0) | 23,036.1 (5.0) | 0.009 |
| COPD | 30,233.5 (6.2) | 5,250.3 (6.1) | 27,175.9 (5.8) | 0.007 |
| Pneumonia | 27,732.5 (5.7) | 4,664.2 (5.4) | 23,830.3 (5.1) | 0.007 |
| Psychiatric disorder |  |  |  |  |
| Depression | 20,902.4 (4.3) | 3,709.8 (4.3) | 19,544.5 (4.2) | 0.017 |
| Anxiety | 42,544.1 (8.8) | 7,544.4 (8.8) | 40,674.1 (8.7) | 0.027 |
| Schizophrenia | 4,836.9 (1.0) | 839.5 (1.0) | 4,772.2 (1.0) | 0.006 |
| Neurologic disorders |  |  |  |  |
| Dementia | 17,600.6 (3.6) | 3,106.1 (3.6) | 14,432.8 (3.1) | 0.006 |
| Epilepsy | 4,047.1 (0.8) | 732.5 (0.9) | 3,677.2 (0.8) | 0.006 |
| Bone and joint disorders |  |  |  |  |
| Fracture | 27,073.6 (5.6) | 4,836.0 (5.6) | 25,158.4 (5.4) | 0.029 |
| Osteoporosis | 11,108.4 (2.3) | 2,155.0 (2.5) | 10,388.7 (2.2) | 0.006 |
| Osteoarthritis | 75,821.2 (15.7) | 13,598.5 (15.9) | 72,027.8 (15.5) | 0.008 |
| Anemia | 19,628.9 (4.1) | 3,532.8 (4.1) | 17,739.9 (3.8) | 0.005 |
| Thyroid disease | 20,998.9 (4.3) | 3,690 (4.3) | 19,816.3 (4.3) | 0.005 |
| Liver disease | 57,709.7 (11.9) | 10,159.2 (11.9) | 56,412.9 (12.1) | 0.012 |
| Chronic kidney disease | 84,727.6 (17.5) | 15,228.3 (17.8) | 75,994.5 (16.3) | 0.005 |
| Hyperkalemia | 2,792.3 (0.6) | 369.2 (0.4) | 1,792.9 (0.4) | 0.007 |
| Hypokalemia | 4,824.7 (1.0) | 787.3 (0.9) | 3,973.7 (0.9) | 0.031 |
| Autoimmune diseases | 11,596.8 (2.4) | 2,082.3 (2.4) | 10,664.7 (2.3) | 0.028 |
| Cancer | 18,571.7 (3.8) | 3,228.8 (3.8) | 17,171 (3.7) | 0.015 |
| GERD | 35,235.2 (7.3) | 6,376.3 (7.4) | 33,635.9 (7.2) | 0.007 |
| **Co-medication, No. (%)** |  |  |  |  |
| Cardiovascular comedication | |  |  |  |
| ACEIs | 29,303.0 (6.1) | 5,269.1 (6.2) | 27,628.5 (5.9) | 0.008 |
| ARBs | 182,690.0 (37.7) | 32,897.4 (38.4) | 173,410.0 (37.3) | 0.003 |
| Alpha-blockers | 3,640.8 (0.8) | 612.1 (0.7) | 3,387.2 (0.7) | 0.005 |
| Beta-blockers | 132,844.0 (27.4) | 23,515.2 (27.4) | 123,476.0 (26.5) | 0.009 |
| Calcium channel blockers |  |  |  |  |
| Dihydropyridines | 184,395.0 (38.1) | 32,869.3 (38.3) | 173,645.0 (37.3) | 0.003 |
| Non-dihydropyridines | 1,7645.9 (3.6) | 2,913.8 (3.4) | 15,828.5 (3.4) | 0.020 |
| Diuretics |  |  |  |  |
| Thiazides | 71,936.4 (14.9) | 13,173.6 (15.4) | 68,722.3 (14.8) | 0.015 |
| Loop | 44,946.0 (9.3) | 7,851.5 (9.2) | 37,764.5 (8.1) | 0.013 |
| Potassium-sparing agents | 15,849.6 (3.3) | 2,913.1 (3.4) | 13,993.9 (3.0) | 0.003 |
| Antiplatelets | 118,611.0 (24.5) | 20,942.9 (24.4) | 108,748.0 (23.4) | 0.041 |
| Anticoagulants | 21,151.0 (4.4) | 3,471.3 (4.1) | 16,988.9 (3.7) | 0.015 |
| Statins | 183,448.0 (37.9) | 33,268.9 (38.8) | 176,487.0 (37.9) | 0.026 |
| Others lipid-lowering agents | 38,227.1 (7.9) | 6,856.9 (8.0) | 37,292.7 (8.0) | 0.036 |
| Nitrates | 36,029.7 (7.4) | 6,059.2 (7.1) | 31,357.5 (6.7) | 0.002 |
| Antiarrhythmics | 16,352.0 (3.4) | 2,832.0 (3.3) | 13,865.4 (3.0) | 0.005 |
| Digoxin | 6,458.3 (1.3) | 1,161.5 (1.4) | 5,833.4 (1.3) | 0.027 |
| Erythropoietin Stimulating Agents | 11,243.7 (2.3) | 1,623.3 (1.9) | 7,176.0 (1.5) | 0.023 |
| Anti-inflammatory agents |  |  |  |  |
| NSAIDs | 264,951.0 (54.7) | 47,277.0 (55.2) | 255,332.0 (54.9) | 0.007 |
| Steroids | 101,084.0 (20.9) | 18,046.4 (21.1) | 94,835.5 (20.4) | 0.057 |
| K channel opener | 10,013.3 (2.1) | 1,670.1 (2.0) | 8,872.9 (1.9) | 0.004 |
| Inhibitors of mitochondrial PT pore | 130,176.0 (26.9) | 23,049.3 (26.9) | 120,874.0 (26.0) | 0.012 |
| Proton pump inhibitors | 42,344.6 (8.7) | 7,294.4 (8.5) | 37,545.7 (8.1) | 0.012 |
| Anticonvulsants | 40,318.8 (8.3) | 6,838.8 (8.0) | 35,945.0 (7.7) | 0.020 |
| Antidepressants | 41,002.2 (8.5) | 7,234.0 (8.4) | 37,753.7 (8.1) | 0.024 |
| Antipsychotics | 46,945.2 (9.7) | 8,112.1 (9.5) | 42,376.9 (9.1) | 0.022 |

Abbreviations: DPP-4 inhibitors, dipeptidyl peptidase-4 inhibitors; MitoK_ATP_, mitochondrial ATP-sensitive potassium channel; IPTW, inverse probability of treatment weighting; aSMD, absolute standardized mean difference; No, number; SD, standard deviation; DM, diabetes mellitus; NTD, New Taiwan dollar; aDSCI, adapted Diabetes Complications Severity Index; SGLT2i, sodium-glucose co-transporter 2 inhibitor; HbA1c, glycated hemoglobin; COPD, chronic obstructive pulmonary disease; GERD, gastroesophageal reflux disease; ACEIs, angiotensin converting enzyme inhibitors; ARBs, angiotensin receptor blockers; NSAIDs, nonsteroidal anti-inflammatory drugs; PT, permeability transition.

^*^ All co-medications, diabetes severity indicators, health care utilization, and monthly income were measured in the year preceding the cohort entry date; all comorbidities and diabetes drugs in baseline were measured 180 days preceding the cohort entry date; age, sex, entry year, and hospital level were measured at the cohort entry date.

^†^ Standardized mean difference >0.1 represents meaningful differences between two groups.

^‡^Use of GLP-1RA was not considered in the propensity score estimation model because GLP-1 RA was not allowed to be concomitantly used with DPP-4i based on the reimbursement policy of Taiwan’s national health insurance program.

**e-Table 6. The patient-based number needed to harm for the comparative results across three groups**

| **Outcome** | **3-point MACE** | | | **MI** | | |
| --- | --- | --- | --- | --- | --- | --- |
|  | Cumulative  incidence* | Cumulative  incidence difference | NNH* | Cumulative  incidence* | Cumulative  incidence difference | NNH* |
| **MitoK_ATP_ high-affinity SU *vs.***  **DPP-4i** | 0.0209 | 0.0038 | 263 | 0.0036 | 0.0005 | 2000 |
|  | 0.0171 |  |  | 0.0031 |  |  |
|  | **Ischemic stroke** | | | **CV death** | | |
|  | Cumulative  incidence* | Cumulative  incidence difference | NNH* | Cumulative  incidence* | Cumulative  incidence difference | NNH* |
| **MitoK_ATP_ high-affinity SU *vs.***  **DPP-4i** | 0.0094 | 0.0015 | 667 | 0.0169 | 0.0029 | 345 |
|  | 0.0079 |  |  | 0.0140 |  |  |
|  | **Hypoglycemia** | | | **All-cause mortality** | | |
|  | Cumulative  incidence* | Cumulative  incidence difference | NNH* | Cumulative  incidence* | Cumulative  incidence difference | NNH* |
| **MitoK_ATP_ low-affinity SU *vs.***  **DPP- 4i** | 0.0025 | 0.0014 | 714 | NA | NA | NA |
|  | 0.0011 |  |  |  |  |  |
| **MitoK_ATP_ high-affinity SU vs**  **DPP-4i** | 0.0034 | 0.0023 | 435 | 0.0467 | 0.0129 | 78 |
|  | 0.0011 |  |  | 0.0338 |  |  |

Abbreviations: MitoK_ATP_, mitochondrial ATP-sensitive potassium channel; SU, sulfonylurea; DPP-4i, Dipeptidyl peptidase 4 inhibitors; 3-point MACE, 3-point major cardiovascular events outcome; NNH, number needed to harm; MI, Myocardial infarction; CV, cardiovascular; HF, heart failure; NA, not applicable.

*The cumulative incidence for the outcome was obtained from the Kaplan-Meier curves, and the NNH was then calculated by the formula: NNH = 1/ (CL_comparator_– CL_treatment_). NNH was calculated only for comparative safety results with statistical significance. NNH represents the average number of patients who receive MitoK_ATP_ low-affinity SU or MitoK_ATP_ high-affinity rather than DPP-4i for 1 year to prevent one event of the outcome.

# e-Figure 1. Study flow diagram illustrating the selection of users of MitoK_ATP_ channel-low affinity sulfonylureas, MitoK_ATP_ channel-high affinity sulfonylureas, and DPP-4 inhibitors

Abbreviations: MitoK_ATP,_ mitochondrial ATP-sensitive potassium channel; DPP-4 inhibitors, Dipeptidyl peptidase 4 inhibitors.

**(A)** **(B)**

Log-rank *P*= 0.0331

Log-rank *P*< 0.0001

**(C) (D)**

Log-rank *P*< 0.0001

Log-rank *P*= 0.0002

e-Figure 2. Kaplan-Meier survival curves of 3-point MACE (A), MI (B), Ischemic stroke (C), and CV death (D) among three groups

Abbreviations: MitoKATP, mitochondrial ATP-sensitive potassium channel; SU, sulfonylurea; DPP-4 inhibitors, Dipeptidyl peptidase 4 inhibitors; 3-point MACE, 3-point major cardiovascular events outcome; MI, Myocardial infarction; CV, cardiovascular.

 **(A)** **(B)**

Log-rank *P*< 0.0001

Log-rank *P*< 0.0001

**(C) (D)**

Log-rank *P*< 0.0001

Log-rank *P*= 0.03

# e-Figure 3. Kaplan-Meier survival curves of HF (A), Arrhythmia (B), Hypoglycemia (C), and All-cause mortality (D) among three groups

Abbreviations: MitoKATP, mitochondrial ATP-sensitive potassium channel; SU, sulfonylurea; DPP-4 inhibitors, Dipeptidyl peptidase 4 inhibitors; HF, heart failure.

# e-Methods 1. Analysis of patients with metformin monotherapy before adding sulfonylureas or DPP-4 inhibitors.

In order to control the disease severity of T2DM as well as to mitigate potential time-lag bias, we additionally conducted an analysis in the sensitivity analyses that confined T2DM patients with initiation of metformin monotherapy, among whom mitoK_ATP_ channel-low affinity sulfonylureas, mitoK_ATP_ channel-high affinity sulfonylureas, or DPP-4 inhibitors were subsequently added to metformin monotherapy. In comparison with the original cohort, the covariates regarding prior use of other antidiabetic medications were not considered in this redefined cohort as these patients did not have any prescription refill records of other antidiabetic medications in the year before the cohort entry date as well as on the cohort entry date. The results of the redefined cohort of these patients regarding the risk of 3-point MACE showed consistent findings, indicating the robustness of our study findings.
